# Eco-Physiological and Molecular Roles of Zinc Oxide Nanoparticles (ZnO-NPs) in Mitigating Abiotic Stress: A Comprehensive Review

**DOI:** 10.3390/plants15010147

**Published:** 2026-01-04

**Authors:** Erick H. Ochoa-Chaparro, Luis U. Castruita-Esparza, Esteban Sánchez

**Affiliations:** 1 Food and Development Research Center, A.C., Avenida Cuarta Sur No. 3820, Fraccionamiento Vencedores del Desierto, Delicias 33089, Chihuahua, Mexico; eriicktronik@hotmail.com; 2 Faculty of Agricultural and Forestry Sciences, Universidad Autónoma de Chihuahua (UACH), Km. 2.5 Carretera a Rosales, Poniente, Delicias 33000, Chihuahua, Mexico; lcastruita@uach.mx

**Keywords:** oxidative stress regulation, photosystem II efficiency, osmotic adjustment, nanofertilizers safety, climate-smart agriculture

## Abstract

Mitigation of abiotic stress of crops is currently one of the primary issues for modern agriculture to secure food supply. On that point, it is acknowledged that climate change is leading to an increase in temperature and solar radiation, while also contributing to prolonged drought events. In contrast, saline soil and heavy metal pollution have been globally problematic, affecting a large part of crops. In this review, we have provided an overview of the eco-physiological and molecular aspects of zinc oxide nanoparticles (ZnO-NPs) as a novel technology for alleviating abiotic stress in plants. It is reported that the presence of ZnO-NPs has positive benefits in physiological processes, such as photosynthetic efficiency, osmotic regulation, ionic homeostasis, and the activation of antioxidant defense systems through gene modifications and the regulation of genes that are regulated under stress conditions. These are positive results for yields, nutrition, and resistance levels in cereals, legumes, and horticultural crops. Furthermore, essential details are reported, suggesting that the addition of ZnO-NPs to crops may be involved in regulating plant metabolism. Nonetheless, we recognize that this technology poses significant challenges for validation on a large scale, particularly in uncontrolled environments.

## 1. Introduction

Climate change represents one of the most complex challenges facing agriculture today. It can significantly affect crops, aggravated by abiotic stress factors such as drought, saline soils, extreme heat, and, in some agroecosystems, the accumulation of heavy metals. These conditions are highly likely to negatively impact crop growth and yield, thus posing a challenge to food security and sustainability [1,2,3,4,5]. These stressors can affect photosynthesis, disrupt osmotic regulation, limit nitrogen assimilation and oxidation-reduction processes, and affect the adaptability of crops to environmental conditions [6,7,8,9]. Fertilizer application methods and genetic improvement programs are only partial solutions to the problem. Most traditional methods are limited by nutrient loss, low utilization efficiency, and environmental risks [10].

Nanotechnology has emerged as a promising approach to mitigate abiotic stress in sustainable farming. ZnO-NPs and other nanomaterials have garnered considerable attention in this area. They exhibit different physical and chemical reactivity compared to bulk forms due to their small size (less than 100 nm) and high surface-to-volume ratio [11,12,13]. When used in controlled Zn delivery systems in agricultural soils, ZnO-NPs have been shown to alter the amount of Zn^2+^ that is available, depending on their synthesis method [14,15]. Moreover, ZnO-NPs have been associated with enhancements in plant metabolic processes, including elevated photosynthetic activity and antioxidative enzyme function, as well as increased resilience to abiotic stressors such as salinity and drought across various species [16,17,18,19,20].

Zinc is an essential micronutrient required for a wide range of physiological and biochemical processes in plants, including enzyme activation, protein synthesis, membrane stability, and hormonal regulation [21]. It plays a central role in photosynthesis and redox homeostasis through its involvement in carbonic anhydrase activity, Cu/Zn-superoxide dismutase, and the stabilization of biomembranes. Consequently, zinc deficiency is commonly associated with reduced photosynthetic efficiency, impaired antioxidant capacity, and increased susceptibility to abiotic stress [22,23]. In this context, the beneficial effects attributed to zinc oxide nanoparticles ultimately rely on the fundamental physiological functions of Zn as an essential element. The nanoscale formulation primarily modulates Zn bioavailability, uptake kinetics, and spatial distribution within plant tissues, thereby influencing the magnitude and timing of Zn-mediated physiological responses under stress conditions.

However, conventional zinc fertilizers often exhibit low bioavailability due to limited plant uptake and substantial losses in certain soil types. ZnO-NPs have therefore attracted considerable interest as an alternative strategy, as they can enhance zinc bioavailability and facilitate its integration into physiological and molecular pathways in plants [24,25,26]. Several studies have demonstrated that ZnO-NP-based formulations, including polymeric compounds, facilitate controlled zinc release in soils, offering a more stable and efficient source than traditional zinc fertilizers [14].

Drought is one of the significant factors that hinders food production worldwide. ZnO-NPs are also known to improve drought tolerance through osmotic adjustment, increased proline levels, and the activation of antioxidants such as SOD (superoxide dismutase) and CAT (catalase) when functionalized with different osmoprotectants, including proline or betaine [19,27,28]. Under drought conditions, ZnO-NPs enhance seedling growth, water status, nutrient uptake, and yield in wheat and Coriandrum sativum [17,29].

Another significant issue affecting crop productivity is soil salinity. ZnO-NPs mitigate salt stress by equilibrating K^+^ and Na^+^ concentrations, maintaining chlorophyll levels, and ensuring the optimal functioning of photosystem II (PSII) [18,20,30]. When ZnO-NPs are mixed with other nanoparticles, such as SiO_2_, they work together to enhance the body’s ability to combat free radicals and facilitate its adaptation to dry and salty conditions [31,32].

The accumulation of heavy metals in soil remains a significant issue for the safe cultivation of crops. ZnO-NPs lessen oxidative stress, keep ionic balance stable, and promote metal chelation and detoxification processes. This helps plants grow more effectively and absorb more nutrients [33,34,35]. Recent reviews [36,37] have consistently emphasized that they serve two functions: they supply micronutrients and aid in stress reduction.

The expected heat stress from global warming also poses a risk to the yields of important crops. ZnO-NPs maintain the ultrastructure of chloroplasts, stabilize their interiors, improve electron transport efficiency, and promote photosynthesis at elevated temperatures [38,39,40,41].

ZnO-NPs are important molecular regulators because they change the expression of stress-responsive genes, increase the transcription of antioxidant enzymes, and affect hormone-related signaling pathways like abscisic acid (ABA), cytokinins, and auxins [16,42,43]. Evidence supports their role in maintaining genomic stability and stress-related signaling, as well as their synergistic interactions with other nanoparticles or bioactive molecules that enhance plant stress tolerance [44,45,46]. Although significant progress has been made, many aspects remain unknown.

We do not know how ZnO-NPs will affect soil microbiota, trophic transfer, and food safety overall, and it is challenging to ensure that all particles are the same size, dose, and delivery method. Moreover, most findings derive from controlled settings rather than field studies [47,48]. To address these deficiencies, omics-based methodologies (transcriptomics, proteomics, and metabolomics) require significant enhancement to elucidate molecular mechanisms and ecological risks [49,50,51]. While ZnO-NPs exhibit significant potential for mitigating biotic stress, most of the scientific literature has focused on their function in relation to abiotic stressors.

This review combines eco-physiological and molecular perspectives on the use of ZnO-NPs in abiotic stressors of drought, salinity, heavy metal contamination, and heat stress. It does this to identify knowledge gaps and suggest new areas of research that could contribute to sustainable agriculture in a changing climate.

## 2. Climate Change, Abiotic Stress, and Plant Eco-Physiology

Climate change is not only a gradual shift in climate norms but also a multiplier of abiotic stresses, intensifying drought, salinity, heavy metal toxicity, and extreme heat events. From an eco-physiological perspective, these overlapping stressors compromise fundamental plant processes such as photosynthesis, stomatal regulation, osmotic adjustment, nutrient assimilation, and redox homeostasis, reducing crop resilience and productivity [1,2,3,6,11].

Under chronic drought conditions, plants experience hydraulic limitations that induce stomatal closure, restricting CO_2_ diffusion and increasing excitation pressure within the photosynthetic apparatus. This imbalance enhances the generation of reactive oxygen species (ROS), impairs photosynthetic efficiency, and activates complex signaling cascades involving phytohormonal regulation and root system remodeling [52,53,54].

Saline stress imposes simultaneous osmotic and ionic constraints; excessive Na^+^ and Cl^−^ accumulation interferes with K^+^ and Ca^2+^ uptake, disrupts ion homeostasis, and promotes oxidative damage to cellular membranes and proteins [7,18,55]. At the molecular level, epigenetic mechanisms including DNA methylation, histone modifications, and miRNA-mediated regulation play a vital role in reprogramming gene expression and enhancing adaptive capacity under saline conditions [56,57,58].

Heat stress further destabilizes eco-physiological performance by impairing the photosynthetic machinery, altering electron transport dynamics, increasing non-photochemical energy dissipation, and constraining carbon assimilation. These effects are strongly associated with oxidative imbalance and the reduced efficiency of PSII under high-temperature regimes [37,59,60].

On the other hand, heavy metals such as Cd, Pb, and Ni disrupt redox homeostasis and provoke oxidative injury to chloroplasts, membranes, and enzymes, with ROS-mediated toxicity representing a central mechanism of damage [35,36,37]. Increasing evidence highlights the relevance of epigenetic strategies in enhancing tolerance to both thermal and metal stress by reprogramming stress-responsive gene networks and chromatin states [58,61,62].

These stressors rarely occur in isolation. Drought can exacerbate the effects of salinity, heat can intensify oxidative damage, and metal toxicity can compromise metabolic resilience. Integrative analyses of combined stresses emphasize the role of feedback loops involving soil degradation, nutrient cycle disruption, and microclimate alteration, which further constrain plant performance under climate change scenarios [1,3,6]. Moreover, the phenomenon of stress memory demonstrates how prior exposure to abiotic stress can prime subsequent responses through the establishment of persistent epigenetic marks and transcriptional preparedness [56,57,58,62].

Climate change functions not only as an incremental stressor but also as a catalyst that intertwines multiple abiotic pressures, pushing plants toward their eco-physiological limits, including reduced carbon fixation, ionic imbalance, oxidative damage, membrane instability, enzyme inhibition, and altered signaling. Understanding these interactions is therefore essential for developing mitigation strategies that simultaneously target multiple vulnerable nodes within the plant system [24,63,64].

That is why innovative solutions are required to enhance plant resilience. Among emerging tools, ZnO-NPs stand out for their eco-physiological and molecular benefits, including improvements in photosynthetic performance, osmotic adjustment, antioxidant defense, and stress-responsive regulation across diverse crops and abiotic stress scenarios [11,41,65,66]. Accordingly, the following section will examine their properties and potential applications in sustainable agriculture.

## 3. ZnO-NPs: Properties and Potential

### 3.1. Physicochemical Properties of ZnO-NPs

ZnO-NPs are characterized by their nanoscale size (<100 nm), high surface-to-volume ratio, and chemical stability, properties that favor controlled dissolution and a gradual release of Zn^2+^ ions under physiological and edaphic conditions. These characteristics enable a more sustained micronutrient supply and reduce losses due to leaching or precipitation compared to conventional bulk Zn formulations [13]. Furthermore, synthesis routes and surface modifications, including green synthesis, coatings, and functionalization, can significantly modulate particle reactivity, dissolution kinetics, and biological interactions [11].

### 3.2. Influence of ZnO-NP Surface Chemistry on Zn^2+^ Availability, Redox Balance, and Photoprotection

Zinc oxide nanoparticles should not be considered a uniform group, as surface chemistry strongly determines their dissolution behavior, Zn^2+^ bioavailability, cellular uptake pathways, and redox interactions. Bare ZnO-NPs typically exhibit higher dissolution rates, leading to increased Zn^2+^ release in the apoplast or cytosol, which can enhance micronutrient availability but also elevate the risk of excessive ROS generation under high concentrations or prolonged exposure. In contrast, surface coatings or functionalization (e.g., polymers, organic ligands, or biogenic layers) can moderate dissolution kinetics, alter nanoparticle–membrane interactions, and shift internalization routes, thereby influencing the balance between ROS production and photoprotective responses.

Importantly, this heterogeneity implies that mechanistic interpretations derived from the literature are not equally robust across nanoparticle types. Therefore, “general” ZnO-NPs mechanisms should be viewed as context-dependent hypotheses that may differ quantitatively—and in some cases qualitatively—between bare and functionalized formulations, depending on dissolution propensity, plant exposure route, and exposure duration [67,68,69].

These surface-dependent differences have direct implications for photosynthetic regulation, particularly non-photochemical quenching (NPQ). Controlled Zn^2+^ availability and moderate ROS signaling may promote adaptive NPQ activation and redox acclimation, whereas excessive Zn^2+^ release or uncontrolled ROS accumulation can overwhelm photoprotective capacity and lead to photoinhibition. Consequently, the interpretation of ZnO-NP-mediated stress tolerance should explicitly consider nanoparticle surface properties, in addition to concentration, exposure duration, and plant species, to avoid overgeneralization [69,70].

### 3.3. Comparison with Conventional Sources of Zn

Compared with soluble or bulk Zn fertilizers, ZnO-NPs have demonstrated enhanced efficiency in Zn uptake and internal translocation. In rice, the application of ZnO-NPs increased Zn accumulation in aerial tissues and improved root-to-shoot transport compared to ionic Zn sources, indicating reduced soil fixation and enhanced mobility within the plant system [71]. These findings suggest that ZnO-NPs function not only as a Zn source but also as effective micronutrient carriers that optimize Zn bioavailability and distribution.

### 3.4. Absorption and Transport Mechanisms

ZnO-NPs can be absorbed through both root and foliar pathways. Once internalized, partial nanoparticle dissolution results in the release of Zn^2+^ ions, which are subsequently transported through the xylem to leaves and other sink tissues [15]. Nanoparticle size plays a critical role in absorption efficiency and physiological impact, as demonstrated in *Agrostis stolonifera*, where different ZnO-NP sizes (30, 50, and 90 nm) produced distinct uptake patterns and metabolic responses [72]. Additionally, in *Phaseolus vulgaris*, in vivo tracking using spectroscopic approaches has confirmed the uptake, storage, and translocation of ZnO-NPs within plant tissues [15].

### 3.5. Environmental and Safety Considerations

Despite their agronomic potential, ZnO-NPs pose environmental and physiological risks when applied at excessive doses. High exposure levels may induce oxidative stress, disrupt soil microbial communities, or cause phytotoxic effects. In lettuce exposed to cadmium stress, ZnO-NPs enhanced antioxidant defense and reduced metal accumulation; however, beneficial effects were strictly dose-dependent, with higher concentrations increasing the risk of cellular damage [33]. Recent reviews emphasize that the efficacy and safety of nanoparticles are strongly influenced by size, concentration, surface chemistry, and stability, and that uncontrolled application can impair membrane integrity and metabolic homeostasis [73].

Beyond ecotoxicological concerns, the physiological impact of ZnO-NPs is intrinsically linked to their interaction with plant cellular processes. Their ability to modulate redox balance, photosynthetic performance, nutrient metabolism, and hormonal signaling underscores the need for safety assessments to be integrated with a mechanistic understanding of these processes. Consequently, a detailed investigation of how ZnO-NPs reprogram antioxidant systems, regulate photosystem II efficiency, influence carbon and nitrogen metabolism, and activate signaling cascades at biochemical and transcriptomic levels is essential. These aspects are addressed in the following section, which discusses the physiological and molecular mechanisms underlying ZnO-NP-induced stress tolerance.

The fate of ZnO-NPs in soil is governed by a complex interplay of physicochemical and biological processes, including aggregation, partial dissolution, adsorption to clay minerals, interaction with soil organic matter, and microbial activity [67,68]. Following soil application, ZnO-NPs may undergo gradual dissolution, releasing Zn^2+^ ions that contribute to plant nutrition, while the remaining particulate fraction can persist or aggregate depending on soil pH, ionic strength, and organic matter content. Acidic soils generally promote the dissolution of ZnO-NPs, increasing Zn^2+^ availability, whereas neutral to alkaline conditions favor aggregation and reduced mobility [74]. Additionally, soil microorganisms can influence nanoparticle transformation and redox behavior, potentially altering both zinc bioavailability and microbial community structure. These dynamics suggest that the agronomic effectiveness and environmental safety of ZnO-NPs are strongly dependent on soil conditions, highlighting the need for site-specific application strategies to balance micronutrient supply with ecological risk [67,74].

## 4. Physiological and Molecular Mechanisms of Tolerance Induced by ZnO-NPs

### 4.1. Regulation of ROS and Antioxidant Systems Using ZnO-NPs

Abiotic stress, including salinity, drought, heavy metal toxicity, and high temperatures, disrupts cellular redox homeostasis in plants by altering the production of reactive oxygen species (ROS) such as superoxide (O_2_^−^), hydrogen peroxide (H_2_O_2_), and hydroxyl radicals (OH•). While excessive ROS accumulation can lead to lipid peroxidation, DNA damage, protein oxidation, and organelle dysfunction, controlled ROS generation also plays a central role in stress signaling, photoprotection, and acclimation processes. In this context, ROS function not only as damaging agents but also as key redox signals that modulate antioxidant defenses and gene expression under abiotic stress [75,76].

To counteract this damage, plants activate a highly regulated antioxidant network that includes enzyme systems such as superoxide dismutase (SOD), catalase (CAT), ascorbate peroxidase (APX), glutathione peroxidase (GPX), peroxidase (POD), and glutathione reductase (GR), as well as non-enzymatic antioxidants such as ascorbic acid (AsA), reduced glutathione (GSH), flavonoids, carotenoids, and tocopherols [54,64].

However, under conditions of intense or prolonged stress, these natural mechanisms may prove insufficient. Several studies have shown that the application of ZnO-NPs enhances the antioxidant response of plants through multiple mechanisms: they stimulate the activity of key enzymes, reduce the accumulation of ROS, and improve redox homeostasis, thus contributing to greater tolerance to environmental stress.

For example, Mishra et al. (2023) [63] demonstrated that ZnO-NPs significantly increase the activity of SOD, CAT, and APX in *Brassica juncea* exposed to heavy metal stress, while simultaneously reducing lipid peroxidation (MDA) levels. Similarly, Gupta et al. (2024) [77] demonstrated that the application of ZnO-NPs under saline stress significantly improves antioxidant enzyme activity (SOD, CAT), proline content, and ionic homeostasis, contributing to improved stress tolerance. In addition to their direct enzymatic action, ZnO-NPs also appear to modulate redox signaling pathways.

For instance, Singh et al. (2025) [46] reports that the foliar application of ZnO-NPs significantly elevated non-enzymatic antioxidants, such as glutathione (GSH) and ascorbate (AsA), in stressed plants, thereby contributing to a more robust ROS-scavenging system and redox homeostasis. These molecules not only detoxify peroxides but also serve as signaling intermediates, allowing plants to fine-tune gene expression in response to oxidative stress.

Furthermore, evidence from Ali et al. (2025) [45] demonstrates that ZnO-NPs regulate the redox state of glutathione by increasing GSH levels and improving the GSH/GSSG ratio in *Brassica napus*, suggesting enhanced cellular reducing power. This shift in redox potential is crucial in maintaining protein thiol integrity and preventing irreversible oxidative damage.

These findings consolidate the role of ZnO-NPs not only as passive antioxidants but also as active modulators of cellular redox networks, with implications for transcriptional regulation, stress signaling, and enhanced physiological resilience under abiotic stress conditions.

### 4.2. Photosynthesis and PSII

Abiotic stress, including drought, salinity, extreme heat, and heavy metal toxicity, profoundly affects the efficiency of photosystem II (PSII), a key component of photosynthesis in plants. These adverse conditions alter critical parameters such as the maximum quantum efficiency of PSII (Fv/Fm), electron transport rate, and overall photosynthetic efficiency. For example, studies have shown that salt and heat stress significantly reduce the activity of the PSII reaction center, preventing the repair of essential proteins such as D1 and causing the accumulation of reactive oxygen species (ROS), which exacerbates damage to thylakoid membranes and decreases quantum yield [52,60]. In rice and young apple trees, drought and salinity have been shown to cause a decrease in PSII energy conversion efficiency of up to 76%, as measured by Fv/Fm, as well as a collapse in the electron transport chain [53].

For example, Elshoky et al. (2021) [78] studied *Pisum sativum* under salt stress and demonstrated that foliar application of ZnO-NPs significantly improved Fv/Fm, ΦPSII, and qp values, indicating a restoration of PSII function. In addition, the negative impact of NaCl on PSI (P700) photooxidation was reduced, suggesting that nanoparticles also protect downstream electron flow.

Complementarily, Seleiman et al. (2023) [79] observed in *Zea mays* grown under saline conditions that treatment with ZnO-NPs not only maintained higher PSII quantum efficiency (higher Fv/Fm values) but also prevented the collapse of electron transport. This effect was accompanied by increased chlorophyll accumulation and improved stomatal conductance, reflecting better integration of photoprotection with carbon metabolism.

In *Capsicum annuum*, Rasouli et al. (2022) [80] reported that foliar application of ZnO-NPs under saline stress led to significant increases in PSII quantum efficiency and a reduction in Y(NO), a parameter associated with non-photochemical energy dissipation. These responses suggest a more efficient allocation of absorbed light energy toward photochemical processes under ZnO-NPs treatment.

Nevertheless, improvements in individual chlorophyll fluorescence parameters should not be interpreted in isolation as definitive indicators of overall photosynthetic stability. Under certain conditions, enhanced values of Fv/Fm, ΦPSII, or qP may conceal underlying phototoxic effects, including excessive excitation pressure, localized oxidative damage, or destabilization of thylakoid membranes. Therefore, fluorescence-based assessments of ZnO-NPs’ effects on photosynthesis should be interpreted alongside complementary indicators, such as gas exchange, ROS accumulation, and exposure duration, to accurately evaluate long-term photosynthetic performance.

In addition, Azarin et al. (2023) [81] analyzed the expression of PSII-related genes in Hordeum vulgare and demonstrated that plants treated with ZnO-NPs showed increased expression of genes encoding PSII complex subunits, suggesting a transcriptional contribution to PSII repair and functional maintenance under stress.

These studies indicate that ZnO-NP application can modulate PSII-related photochemical parameters under abiotic stress, including Fv/Fm, ΦPSII, and electron transport rates. However, such enhancements should be interpreted with caution, as increased photochemical efficiency may coexist with donor-side photoinhibition, partial impairment of the oxygen-evolving complex, or elevated ROS production under specific dose- and time-dependent exposure scenarios. In this context, apparent improvements in PSII performance may reflect compensatory or transient photophysical adjustments rather than sustained structural protection of the photosynthetic apparatus.

To improve interpretability, it is important to distinguish between short-term photophysical responses and longer-term acclimation processes induced by ZnO-NP exposure. Transient responses, often observed shortly after application, may include rapid adjustments in energy partitioning within PSII, modulation of non-photochemical quenching, or temporary shifts in redox status. In contrast, true physiological acclimation involves sustained modifications, such as the reinforcement of antioxidant systems, stabilization of ion homeostasis, osmotic adjustment, and longer-term changes in gene expression. Failure to distinguish between these temporal scales may lead to the overinterpretation of early photochemical responses as indicators of durable stress tolerance.

### 4.3. Nitrogen and Carbon Metabolism

Nitrogen and carbon metabolism are central to plant growth and are particularly sensitive to abiotic stress conditions such as salinity, drought, heavy metal toxicity, and elevated temperatures. These stressors compromise the activity of key enzymes such as nitrate reductase (NR), glutamine synthetase (GS), and Rubisco, reducing nutrient use efficiency and, consequently, crop yield. For example, reduced NR activity limits nitrogen assimilation, while Rubisco inhibition directly affects carbon fixation, resulting in a decrease in the net photosynthetic rate and subsequent reduction in biomass accumulation [65,82].

The use of ZnO-NPs has been shown to have a positive effect in counteracting these limitations. Several studies have documented that foliar or root application of ZnO-NPs can restore or significantly improve NR and GS activity under abiotic stress, favoring nitrogen assimilation and promoting the biosynthesis of essential amino acids [16].

At the level of carbon metabolism, ZnO-NPs also promote substantial improvements. These nanoparticles have been observed to increase the expression and activity of the Rubisco enzyme, as well as its activase, resulting in enhanced photosynthetic capacity under adverse conditions [65,83]. This metabolic stimulation allows adequate carbon fixation rates to be maintained even under water or salt stress. In addition, ZnO-NPs contribute to a favorable energy balance by improving nutrient use efficiency, which is reflected in greater biomass accumulation and protein content in plant tissues [84,85].

The mechanism of action of ZnO-NPs not only involves a controlled supply of zinc, an essential cofactor for many enzymes involved in these processes, but also an interaction with molecular networks that modulate gene expression associated with primary metabolism. In fact, Mirakhorli et al. (2021) [16] demonstrated that ZnO-NPs activate transcription factors related to the epigenetic regulation of nitrogen and carbon assimilation pathways, such as histone deacetylase-dependent pathways and hormonal signals such as abscisic acid (ABA).

The ZnO-NPs are promising tools for optimizing plant metabolic efficiency, especially in agricultural settings affected by multiple environmental stressors. Their ability to improve the functionality of NR, GS, and Rubisco, as well as to enhance nitrogen and carbon use efficiency, represents a strategic advance toward more resilient and sustainable cropping systems.

### 4.4. Hormonal Signaling

The role of ZnO-NPs in modulating hormonal pathways in plants subjected to abiotic stress has attracted increasing attention in recent years. Phytohormones, such as abscisic acid (ABA), auxins (IAA), and cytokinins, play a crucial role in stress perception and response, as they regulate processes including stomatal opening, leaf senescence, root development, and antioxidant response. Several studies indicate that ZnO-NPs can directly influence the biosynthesis, degradation, or signaling of these hormones, thus modulating the adaptive capacity of plants to adverse conditions such as drought, salinity, or heavy metal toxicity [44,86].

ABA is one of the hormones most involved in the response to water and salt stress. Its accumulation promotes stomatal closure and activates genes related to dehydration tolerance. Recent research has shown that the application of ZnO-NPs can induce an increase in ABA concentration, as well as the expression of ABA-dependent genes, such as RD29A and NCED3, responsible for the synthesis of this hormone [87]. In corn, for example, ABA-loaded ZnO-NPs have been shown to significantly improve drought tolerance by increasing root hydraulic conductivity and reducing foliar water loss [88].

For auxins, it has been observed that the application of ZnO-NPs can alter their concentration and transport. In *Arabidopsis thaliana*, Vankova et al. (2017) [89] reported that high concentrations of ZnO-NPs decrease IAA levels in shoot apices, which can influence growth and branching patterns under stress. However, this modulation can be beneficial, as it can prevent uncontrolled growth and redirect resources toward survival under certain conditions.

On the other hand, cytokinin hormones, which promote cell division and delay senescence, are also sensitive to the presence of ZnO-NPs. In a study using *Phaseolus vulgaris*, Amer (2024) [90] demonstrated that cytokinin biosynthesis increased after the application of green ZnO-NPs (synthesized using plant extracts), accompanied by an increase in the expression of genes such as IPT and CKX. This effect contributed to maintaining leaf viability and prolonging photosynthetic activity during salinity stress.

Sonkar et al. (2021) [91] provides additional evidence of the role of ZnO-NPs in hormone regulation by observing that these particles suppress the production of auxins and cytokinins under stress conditions but induce the accumulation of cis-zeatin, a cytokinin associated with defense responses. This finding suggests that ZnO-NPs not only modify hormone concentrations but also alter the profiles of specific isoforms with differentiated functions.

Tripathi et al. (2022) [86] describe a complex network of interactions between nanoparticles and plant hormones, in which ZnO-NPs act as triggers for hormonal signaling cascades that include not only ABA, IAA, and cytokinins, but also ethylene and jasmonic acid. These interactions are mediated by redox signals, specific receptors, and epigenetic modifications that amplify or repress hormonal responses depending on the type and severity of stress.

Studies such as that by Azhar et al. (2021) [92] have documented that treatment with ZnO-NPs increases the expression of genes related to ethylene signaling and reduces the expression of cytokinin genes in *Arabidopsis*, which could be related to the promotion of senescence as a nutrient recycling strategy under chronic stress.

### 4.5. Genes and Transcriptomics

The role of ZnO-NPs in plants extends beyond immediate physiological effects, manifesting itself in transcriptional reprogramming that modulates key genes associated with antioxidants, ion transport, photosynthesis, hormonal signaling, and metal detoxification. This molecular plasticity is crucial for plant adaptation to multiple abiotic stressors.

One of the most consistent responses is the induction of genes encoding antioxidant enzymes. In tomatoes subjected to salinity, ZnO-NPs were observed to activate the expression of SOD, CAT, and APX, significantly reducing ROS levels and preventing lipid peroxidation [4].

Integrated transcriptomic and metabolomic analyses in tomato demonstrated that ZnO nanoparticles alter the expression of antioxidant-related genes and key secondary metabolic pathways, particularly those associated with phenylpropanoid and flavonoid biosynthesis [93]. Under salinity stress, ZnO-NPs also modulate genes that govern ion transport and cellular ion homeostasis.

The overexpression of NHX and HKT1, involved in Na^+^ exclusion and K^+^ retention, as well as SOS1, which participates in Na^+^ expulsion at the plasma membrane level, has been reported. These changes allow a functional ionic balance to be maintained even under high salinity levels [55].

ZnO-NPs also modulate genes linked to photosynthesis. In *Brassica napus*, proteomic and transcriptomic analyses showed the activation of genes associated with the Calvin cycle, including Rubisco activase (RCA), and PSII structural genes such as psbA, which contributed to sustaining photosynthetic efficiency under oxidative stress [94].

Under drought conditions, the impact of ZnO-NPs is reflected in the activation of key genes in hormonal signaling. Studies have shown that ABA-functionalized nanoparticles increase the expression of NCED3, a key enzyme in ABA biosynthesis, as well as response genes such as RD29A, DREB2A, and AREB1, which are involved in dehydration tolerance and the induction of protective osmolytes [87].

Under conditions of Cd and Pb toxicity, ZnO-NPs promote the expression of genes involved in metal chelation and detoxification, such as PCS1 (phytochelatin synthase) and MT2 (metallothionein), in addition to enhancing the activity of antioxidant genes such as GPX and GR, which are related to the glutathione cycle [16].

Transcriptomic evidence indicates that ZnO-NPs function not only as zinc suppliers but also as molecular modulators, capable of activating distinct sets of genes that vary according to the type of abiotic stress. This dynamic control over transcriptomic networks offers a mechanistic explanation for the ability of ZnO-NPs to improve plant resilience to climate change.

## 5. Experimental Evidence in Crops Under Abiotic Stress

### 5.1. Drought Stress and the Role of ZnO-NPs

Drought is one of the most destructive abiotic stress factors affecting crops, as it alters water balance, impairs photosynthesis, and accelerates oxidative damage. There is growing evidence that ZnO-NPs mitigate these effects by maintaining plant water status, stabilizing photosynthetic pigments, and enhancing enzymatic and non-enzymatic antioxidant defenses.

For example, foliar ZnO-NPs increased relative water content, chlorophyll retention, and yield stability in eggplants and tomatoes exposed to severe drought [95,96], while in wheat they advanced panicle initiation and improved grain yield by more than 40% compared to bulk Zn fertilizers [66]. Similar physiological improvements, such as osmolite accumulation, proline biosynthesis, and restoration of redox balance, have been reported in cucumbers, peas, and cotton [97,98,99].

Beyond individual applications, synergistic strategies have proven particularly effective. Combinations of ZnO nanoparticles with melatonin, mycorrhizal fungi, or fulerenols further improved nutrient uptake, osmotic adjustment, and antioxidant capacity, resulting in superior resilience in strawberries, soybeans, and *Arabidopsis* [100,101,102].

Table 1 summarizes these results, highlighting the diversity of crops, application methods, and physiological responses enhanced by ZnO-NPs under drought conditions.

**Figure 1:**
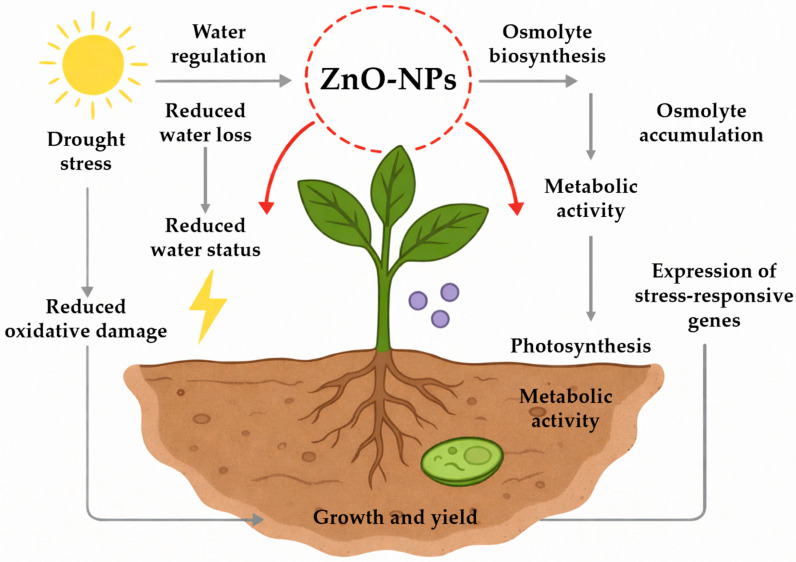
Drought stress and the Role of ZnO-NPs.

The evidence indicates that ZnO-NPs act not only as efficient Zn^2+^ carriers but as nanoregulators of drought tolerance, coordinating water balance, antioxidant activity, and metabolic adjustments (Figure 1). However, most findings derive from controlled environments; field validations, dosage optimization, and long-term ecological assessments remain pressing gaps. Addressing these challenges will be essential to fully harness ZnO-NPs as part of climate-smart strategies for sustainable agriculture.

### 5.2. Salinity Stress and the Role of ZnO-NPs

Salinity is one of the most widespread abiotic stresses, severely affecting crop growth by inducing osmotic imbalance, ionic toxicity, and oxidative stress. Recent studies consistently demonstrate that ZnO-NPs alleviate salt-induced damage by modulating water relations, improving ion homeostasis, and strengthening antioxidant defense systems. For example, in *Phaseolus vulgaris* and *Pisum sativum*, foliar ZnO-NPs improved relative water content, photosynthetic pigments, and enzymatic activity, reducing H_2_O_2_ and malondialdehyde accumulation under 100–200 mM NaCl stress [20,78]. Similarly, in rice and wheat, ZnO-NPs restored photosystem II efficiency, enhanced nutrient uptake, and increased grain yield even under saline soils, outperforming bulk Zn sources [114,115].

Applications in quinoa, cotton, maize, and coffee further highlight their ability to regulate Na^+^/K^+^ ratios, maintain chlorophyll stability, and upregulate stress-related genes, leading to improved biomass, fiber quality, and yield under saline irrigation regimes [98,99,100,101]. Studies also reveal significant benefits of synergistic treatments, where ZnO-NPs combined with SiO_2_, proline, melatonin, or biochar result in superior antioxidant activity, better osmotic adjustment, and stronger metabolic resilience compared to single applications, as reported in lettuce, spinach, and Salvia species [31,116,117].

Despite these reported advantages, increasing evidence suggests that ZnO-based heteronanostructures and synergistic formulations may exhibit time-dependent trade-offs between enhanced physiological performance and redox stability [69,70]. While short-term exposure often leads to improvements in photosynthetic parameters, antioxidant capacity, and stress-related gene expression, prolonged or repeated exposure has been associated with excessive ROS accumulation and redox imbalance in some studies. These observations indicate that early gains in photosynthetic efficiency or metabolic resilience may not always translate into sustained stress tolerance, particularly if antioxidant and photoprotective systems become saturated. Therefore, the effectiveness of ZnO-based heteronanostructures and combined formulations should be interpreted within a temporal framework that considers both short-term benefits and potential long-term redox constraints.

Across the reviewed studies, ZnO-NP-mediated responses consistently display narrow dose-dependent behavior, allowing the distinction of three functional concentration ranges. Low concentrations, typically in the range of a few mg L^−1^ for foliar or hydroponic applications and tens of mg kg^−1^ in soil-based systems, are most frequently associated with beneficial effects, including improved PSII performance, activation of antioxidants, and enhanced stress tolerance. Intermediate concentrations often result in neutral or marginal physiological responses, with limited gains in photosynthetic or metabolic performance. In contrast, higher concentrations—particularly under prolonged or repeated exposure—have been linked to phototoxic outcomes, such as excessive ROS accumulation, photoinhibition, membrane destabilization, and growth suppression. These observations indicate that the effective window for ZnO-NP application is relatively narrow and strongly dependent on exposure duration, nanoparticle properties, and plant species, underscoring the need for precise dose optimization to avoid crossing the threshold from stimulation to toxicity [70].

Given the pronounced dose dependence and narrow effective windows reported for ZnO-NPs, their agronomic feasibility under realistic field conditions remains conditional rather than universal. In controlled environments, precise dose regulation enables the achievement of beneficial physiological responses; however, under field conditions, spatial heterogeneity in soil properties, variable moisture regimes, microbial activity, and environmental fluctuations can alter nanoparticle fate, Zn^2+^ availability, and plant exposure. Consequently, unregulated or large-scale application of ZnO-NPs could increase the risk of crossing the threshold from stimulation to phototoxicity. From an agronomic perspective, the most feasible strategies appear to involve low-dose applications, targeted delivery methods (e.g., seed priming or foliar sprays), and integration with existing nutrient management practices rather than blanket soil amendments. These considerations underscore the necessity of field-scale validation and site-specific optimization before ZnO-NPs can be reliably integrated as climate-smart inputs in agricultural systems. Accordingly, ZnO-NPs should currently be viewed as complementary, rather than standalone, inputs within precision nutrient and stress management frameworks [67,68,70].

Notably, nanopriming strategies have also emerged as effective approaches. Seed treatments with ZnO-NPs improved early vigor, nutrient accumulation, and stress tolerance in mung bean, tomato, and chickpea seedlings under salinity conditions, with clear physiological advantages over conventional priming or bulk Zn [118,119,120]. Similar evidence in pepper and basil demonstrates enhanced shoot and root growth, chlorophyll maintenance, and strong antioxidant activation, confirming ZnO-NPs as versatile tools to enhance salt tolerance [121,122].

A synthesis of these findings is presented in Table 2, which integrates results across species, nanoparticle formulations, application modes, and physiological outcomes.

**Figure 2:**
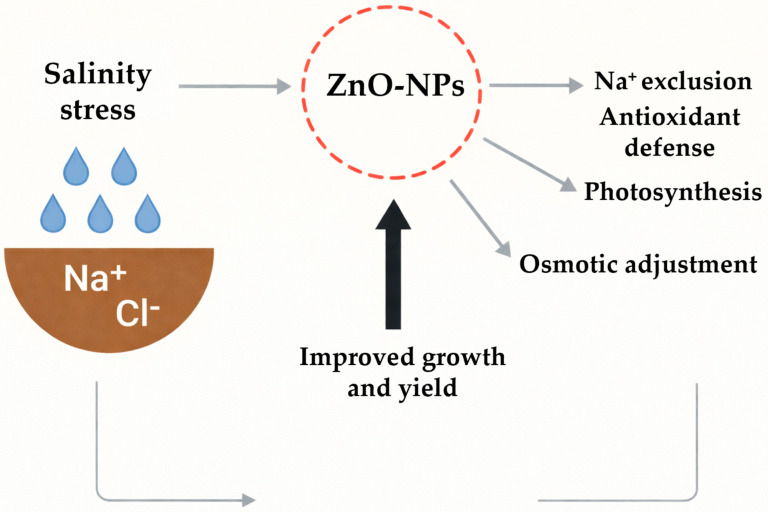
Salinity stress and the role of ZnO-NPs.

The evidence suggests that ZnO-NPs act as multifunctional mitigators of salt stress by enhancing ionic homeostasis, promoting redox balance, and strengthening the photosynthetic machinery (Figure 2). Importantly, their effectiveness increases when combined with other elicitors or nanoformulations, highlighting the potential of integrated strategies. However, despite promising results across legumes, cereals, and horticultural crops, most studies remain restricted to pot or hydroponic conditions, and standardization of dosage, particle size, and delivery methods remain lacking. Future research must bridge this gap with field-scale trials and long-term ecological assessments to consolidate the role of ZnO-NPs in sustainable crop production under salinity stress.

### 5.3. Heat Stress and the Role of ZnO-NPs

Rising global temperatures pose a significant constraint on agricultural productivity, as heat stress impairs photosynthetic performance, accelerates oxidative damage, and destabilizes cellular homeostasis. Recent findings highlight that ZnO-NPs mitigate these effects by enhancing photosynthetic capacity, improving water balance, and strengthening antioxidant defenses. For example, in rice and maize, foliar or seed treatment with ZnO-NPs significantly increased grain yield, chlorophyll stability, and nutrient uptake, while reducing electrolyte loss and enhancing antioxidant enzyme activity under field and controlled heat conditions [40,131].

Similar improvements in chlorophyll retention, carotenoid content, and antioxidant systems were observed in oregano and alfalfa, where ZnO-NPs reduced lipid peroxidation, stabilized membranes, and promoted greater biomass accumulation under prolonged elevated temperature conditions [38,132]. Molecular and metabolic analyses also confirm the role of ZnO-NPs in modulating stress signaling pathways.

In *Arabidopsis thaliana* and *Chlorella*, ZnO-NPs reduced ROS accumulation, restored amino acid and energy metabolism, and prevented heat stress-induced transcriptional silencing, underscoring their role as regulators of redox and gene expression networks [133,134]. In spinach and chickpea, ZnO-NPs not only enhanced enzymatic antioxidants but also acted as nanoprotective agents against combined heat and radiation stress, improving seed germination, vascular integrity, and photoprotection [135,136].

At the physiological level, foliar ZnO-NPs have been particularly effective in stimulating the AsA-GSH cycle, increasing APX, GR, and DHAR activities, and improving proline and soluble sugar accumulation, which together confer greater osmotic adjustment and redox buffering. This was evident in snapdragon and wheat, where 100 mg L^−1^ of foliar ZnO-NPs significantly improved heat tolerance, yield stability, and seed quality, even under conditions of intense heat and combined drought and heat regimes [137,138,139].

Table 3 summarizes these results, compiling evidence from cereals, legumes, oilseeds, vegetables, and model plants, emphasizing the broad protective function of ZnO-NPs at elevated temperatures.

**Figure 3:**
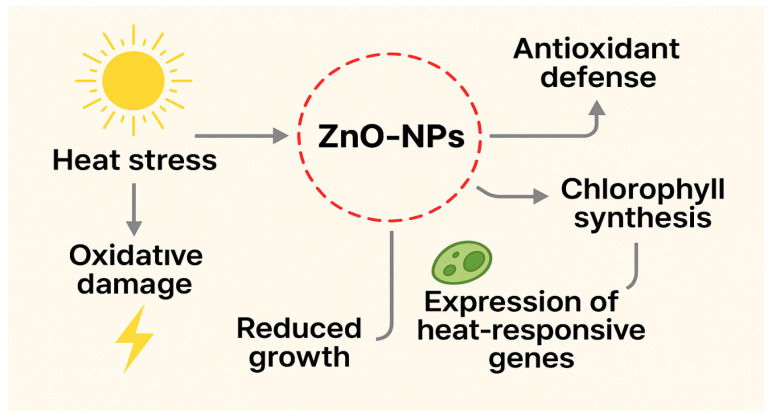
Heat stress and the role of ZnO-NPs.

The studies demonstrate that ZnO-NPs act as multifunctional modulators of heat stress tolerance, improving photosystem efficiency, stabilizing chloroplast ultrastructure, and activating enzymatic and non-enzymatic antioxidants (Figure 3). Evidence also suggests their role in redox-regulated signaling cascades and transcriptional reprogramming, positioning ZnO-NPs as powerful tools for enhancing heat tolerance. However, most experiments have been conducted under controlled conditions, and data on crop performance under prolonged heat waves in the field remain scarce. Future research should focus on field-scale trials, the optimization of nanoparticle delivery, and integrative approaches that combine ZnO-NPs with biostimulants or PGPR to ensure sustainable crop resilience in the context of climate change.

### 5.4. Heavy Metals Stress and the Role of ZnO-NPs

Heavy metal (HM) contamination in agricultural soils is an increasing concern due to its persistence, bioaccumulation, and detrimental effects on plant growth, physiology, and food safety. Cadmium (Cd), lead (Pb), chromium (Cr), and arsenic (As) are particularly toxic, inducing oxidative stress, disrupting membrane integrity, and interfering with nutrient uptake. In this context, ZnO-NPs have emerged as effective agents to mitigate HM toxicity by enhancing antioxidant defense, regulating ion homeostasis, and reducing metal uptake and translocation.

Studies in wheat, maize, and peanuts demonstrate that ZnO-NPs significantly reduce Cd and Cr accumulation, while improving germination, biomass, and photosynthetic pigments [140,141,142]. Transcriptomic evidence further reveals that ZnO-NPs modulate stress-responsive genes, enhance detoxification pathways, and downregulate heavy metal transporters, thereby reducing HM mobility and toxicity [143,144]. In alfalfa and pea, ZnO-NPs decreased ROS accumulation, lipid peroxidation, and programmed cell death, while strengthening osmotic adjustment and enzymatic activity [145,146].

Synergistic strategies have been particularly promising. The co-application of ZnO-NPs with melatonin, biochar, or PGPR amplified protective responses, improved nutrient homeostasis, and enhanced tolerance to Cd, Pb, and Cr stress in tomato, spinach, and mustard [147,148,149]. Likewise, the combination of ZnO with TiO_2_ or Fe_3_O_4_ nanoparticles improved antioxidant activity and reduced Pb uptake in *Echinacea purpurea* and dandelion, highlighting the benefits of hybrid nanocomposites [150,151].

Evidence from metabolomic and ionomics analyses indicates that ZnO-NPs restore key primary metabolites (amino acids, sugars, fatty acids) and regulate rhizosphere microbial networks under HM stress, thereby reinforcing systemic tolerance [152,153]. Such effects confirm that ZnO-NPs act not only as Zn^2+^ sources but also as nano-remediators with dual roles in plant protection and soil detoxification.

A synthesis of these results is presented in Table 4, which integrates evidence across different crops, heavy metal types, nanoparticle formulations, and application modes.

**Figure 4:**
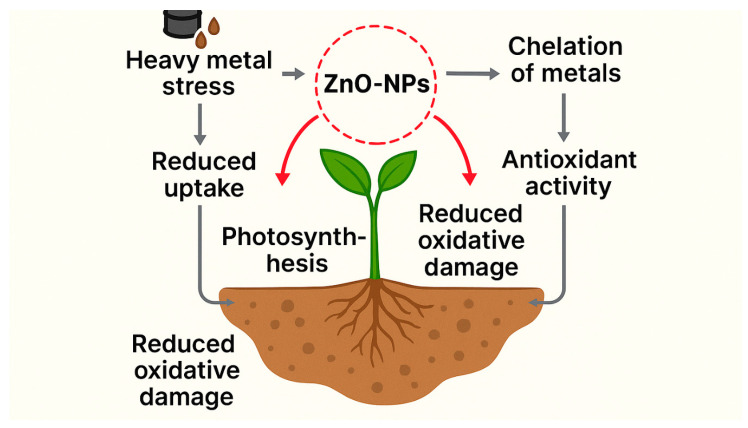
Heavy metals stress and the role of ZnO-NPs.

ZnO-NPs mitigate heavy metal stress by reducing metal uptake and accumulation, activating enzymatic (SOD, CAT, POD, APX) and non-enzymatic antioxidants, and reprogramming metabolic and genetic responses (Figure 4). Their integration with melatonin, biochar, or microbial inoculants further enhances their remediation potential. However, despite convincing evidence from greenhouse and hydroponic experiments, large-scale field validation and risk assessments on food safety and soil microbiota remain limited. Future research should focus on optimizing nanoformulations, ensuring safe application rates, and exploring long-term ecological impacts to consolidate ZnO-NPs as sustainable solutions for managing heavy metal stress in crops.

Collectively, the available evidence suggests that ZnO-NP-mediated stress tolerance cannot be interpreted as entirely stress-specific or fully universal. Across diverse abiotic stresses including salinity, drought, metal toxicity, and thermal stress, recurrent protective responses emerge, such as improved redox homeostasis, stabilization of photosystem II, enhanced antioxidant capacity, and optimized nutrient balance. These shared responses point to the existence of common core pathways modulated by ZnO-NPs. However, the magnitude and downstream manifestation of these pathways are strongly shaped by stress-specific factors, including the primary physiological target of the stress (osmotic, ionic, oxidative, or thermal), exposure duration, and plant species. Thus, ZnO-NPs appear to activate a conserved physiological core that is subsequently fine-tuned according to the specific stress context, rather than eliciting entirely independent mechanisms for each stress type [67,69,70].

The effectiveness of ZnO-NPs varies markedly among plant species, reflecting differences in root system architecture, leaf morphology, nutrient demand, and basal antioxidant capacity. Cereals frequently exhibit great improvements in photosynthetic performance and nutrient use efficiency, whereas legumes and horticultural crops tend to display more pronounced antioxidants and osmotic responses. Such variability has been consistently reported across species and experimental systems, highlighting that ZnO-NPs uptake, translocation, and physiological outcomes are inherently species-dependent and influenced by plant functional traits [69,160].

## 6. Eco-Physiological Role of ZnO-NPs Under Climate Change

### 6.1. Eco-Physiological Perspective in Climate Change Scenarios

Climate change poses an unprecedented challenge to agricultural systems, as it intensifies the frequency of droughts, heat waves, soil salinization, and heavy metal contamination, directly affecting plant physiology and global food security. In this context, nanomaterials, particularly ZnO-NPs, have emerged as an innovative alternative for enhancing crop resilience. Several studies have demonstrated that these particles can modulate the redox balance, enhance photosynthetic efficiency, and improve ionic homeostasis, thereby playing a crucial role in climate-smart agriculture [130,161].

### 6.2. ZnO-NPs as a Tool for Climate Resilience

Eco-physiological tests suggest that ZnO-NPs act as nanoregulators, stabilizing photosynthetic functions under heat and radiation, maintaining membrane integrity in the face of drought and salinity, and inducing faster and more efficient antioxidant responses than conventional zinc sources. In addition, they have been shown to improve nutrient uptake and biofortification under stress conditions, as in the case of soybeans, rice, and coffee subjected to water deficit or saline soils [27,126,128]. Similarly, in the face of toxicity from metals such as lead and cadmium, ZnO nanoparticles show protective effects by reducing oxidative stress and regulating the molecular mechanisms associated with tolerance [146,149,162]. At the molecular level, many interpretations of ZnO-NP-mediated climate resilience are still largely derived from targeted gene-level studies, particularly those focusing on antioxidant enzymes, ion transporters, and stress-responsive regulators. While these approaches provide valuable mechanistic insights, their generalization across species and stress scenarios remains constrained in the absence of broader omics-level validation. Emerging transcriptomic evidence indicates that ZnO-NPs can induce the coordinated reprogramming of redox metabolism, photosynthetic machinery, and secondary metabolic pathways; however, such integrative datasets remain limited and highly context-dependent. Consequently, current molecular interpretations should be regarded as indicative rather than universal, highlighting the need for integrated multi-omics approaches to strengthen causal links between nanoparticle properties, molecular regulation, and whole-plant performance under climate change scenarios [67,69,93].

### 6.3. Possibilities in Protected and Open-Air Agriculture

The application of ZnO-NPs presents numerous opportunities, depending on the cropping system. In controlled environments, such as greenhouses, they enable more precise nanopriming and fertigation strategies, resulting in greater nutrient and water efficiency, as well as reduced environmental risks. In open fields, where climatic and edaphic variability is greater, their application has been shown to improve stress tolerance in legumes, vegetables, and cereals, increasing yields and reducing losses. For example, its foliar use in alfalfa and corn has mitigated the effects of salinity and increased productivity [20,130,163]. Similarly, recent studies on tomatoes, chili peppers, and *Arabidopsis thaliana* confirm its effectiveness under drought and combined stress conditions [102,104,106].

### 6.4. Relationship to Food Security and Sustainability

The link between ZnO-NPs, food security, and sustainability lies in their ability to increase crop yields and nutritional quality while reducing dependence on conventional fertilizers. Their use contributes to the zinc biofortification of cereals and vegetables, a crucial aspect in mitigating micronutrient deficiencies in vulnerable populations [24,35]. When integrated into circular economy schemes and used in synergy with other nanomaterials such as silicon or titanium dioxide, it promotes more sustainable agriculture with a lower environmental footprint [22,150].

In light of the evidence discussed throughout this review, an integrated eco-physiological framework can be proposed to conceptualize ZnO-NP-mediated stress tolerance in plants. Within this framework, the physicochemical properties of ZnO-NPs, particularly size, surface chemistry, and dissolution behavior, govern Zn^2+^ bioavailability and nanoparticle–plant interactions at the cellular interface. These initial interactions modulate redox homeostasis by influencing both ROS production and antioxidant capacity, thereby shaping redox signaling and cellular acclimation. Redox regulation, in turn, plays a central role in stabilizing photosystem II function, affecting photochemical efficiency, electron transport, and photoprotective mechanisms such as non-photochemical quenching. Maintenance of PSII integrity and redox balance ultimately supports downstream physiological processes, including carbon assimilation, nutrient use efficiency, growth, and yield under abiotic stress. This framework emphasizes that ZnO-NPs effects emerge from the coupling of nanoparticle traits with core redox- and photosynthesis-centered regulatory hubs, rather than from isolated responses at a single organizational level, providing a unifying perspective to interpret both beneficial outcomes and context-dependent limitations across plant species and stress conditions [128,164].

## 7. Limitations and Future Challenges

Despite promising evidence, significant limitations remain. Most available studies focus on controlled conditions, while large-scale field validation remains scarce. Without this information, it is not easy to extrapolate laboratory results to diverse agroecological systems [27,104].

Another challenge is the absence of economic studies evaluating the cost-effectiveness of using ZnO-NPs compared to traditional fertilizers. Furthermore, although synergistic effects with other compounds have been documented, combinations, doses, and application methods still need to be optimized to maximize benefits and minimize risks [20,161]. Uncertainty about their long-term ecotoxicological impacts also raises questions, especially regarding persistence in soils, accumulation in edible tissues, and interactions with microbiota [146,162].

Finally, the lack of clear regulatory frameworks and low awareness among farmers and consumers may hinder their social acceptance. Overcoming these challenges requires multidisciplinary approaches that integrate plant physiology, nanotechnology, toxicology, agronomy, and socioeconomics [24,102].

## 8. Conclusions

The accumulated evidence highlights the multifunctional role of ZnO-NPs in alleviating principal abiotic stresses, including drought, salinity, heat, and heavy metal toxicity.

By improving photosynthetic efficiency, regulating ion homeostasis, and activating antioxidant systems, ZnO-NPs act as micronutrient carriers and nanoregulators of stress responses. Their integration into crop management strategies has consistently improved the growth, yield, and nutritional quality of cereals, legumes, and horticultural crops, underscoring their potential to enhance agricultural resilience to climate variability.

However, the transition from experimental promise to practical application requires overcoming several challenges. Standardization of nanoparticle synthesis, optimization of application methods, and validation under diverse field conditions remain pressing needs. Equally important are long-term assessments of environmental fate, food safety, and socioeconomic viability to ensure that nanofertilizers contribute positively to sustainable intensification without unwanted ecological trade-offs.

For instance, ZnO-NPs represent a double-edged innovation: on the one hand, they offer unprecedented opportunities to enhance plant resilience and food security in the context of climate change; on the other hand, they require careful governance, interdisciplinary research, and public engagement to ensure their safe and responsible adoption. As climate-related stresses intensify globally, moving from proof-of-concept studies to integrated and ecologically safe applications of ZnO-NPs will be a decisive step toward achieving climate-smart and sustainable agriculture.

## Figures and Tables

**Table 1:** Effects of zinc oxide nanoparticles (ZnO-NPs) on plant responses under drought stress.

Abiotic Stress	Nanoparticles	Crop	Type of Application and Doses	Response Under the Effect of NPs	Reference
Drought stress (60% ETc) and salinity (saline soil)	ZnO-NPs	Eggplant (*Solanum melongena* L.)	0, 50, 100 ppm (foliar spray)	Increased relative water content, membrane stability, photosynthetic efficiency, growth, yield (12–23%), and water productivity (51–66%).	[95]
Drought (100, 75, 50, 25% field capacity)	Green ZnO-NPs	Tomato (*Solanum lycopersicum* L.)	25, 50, 100 mg L^−1^ (foliar spray)	Improved shoot and root biomass; 25–50 mg/L increased shoot dry weight 2–2.5-fold under severe drought; reduced malondialdehyde and hydrogen peroxide; enhanced antioxidant enzymes (SOD, CAT, APX up to 4.6-, 3.6-, and 3.3-fold higher); 100 mg/L increased oxidative stress.	[96]
Drought (40% field moisture capacity) vs. non-drought (80% FMC)	Zinc-NPs (urea-coated 1%) vs. bulk ZnO (2%)	Wheat (*Triticum aestivum*)	≤2.17 mg kg^−1^ ZnO-NPs, ≤4.34 mg kg^−1^ bulk ZnO	Under drought, ZnO-NPs reduced panicle initiation by 5 days, increased grain yield by 39–51%, and improved Zn uptake by 24%. Bulk ZnO had no significant effect on yield. Nanoparticles achieved higher efficiency with lower Zn input.	[66]
Drought (50% field capacity)	ZnO-NPs	Moringa (*Moringa peregrina*)	0.05% and 0.1% (foliar aerosol)	Prevented chlorophyll degradation, increased chlorophyll content in well-watered plants, enhanced total phenolic content, and antioxidant activity under both drought and non-drought conditions.	[103]
Drought stress (withholding irrigation for 7 days)	Green synthesized and commercial ZnO-NPs	Chili pepper (*Capsicum annuum* L.)	0, 500, 1000 mg L^−1^ (foliar application)	Drought reduced relative water content and leaf water potential while increasing proline, TBARS, and antioxidant enzymes. ZnO-NPs improved water status and reduced oxidative markers; green-synthesized ZnO-NPs (100–500 mg/L) were more effective than chemically synthesized ones in mitigating the effects of drought.	[104]
Drought stress (induced with sorbitol 0.0–0.4 M)	(ZnO-NPs) and magnetite nanoparticles (Fe_3_O_4_-NPs)	Potato (*Solanum tuberosum* L.)	0.0, 2.5, 5.0 mg mL^−1^	Sorbitol ≥ 0.3 M reduced growth and stopped at 0.4 M. ZnO-NPs (2.5–5.0 mg mL^−1^) and Fe_3_O_4_-NPs (2.5–5.0 mg mL^−1^) improved micropropagation, microtuberization, and biochemical traits under drought. Almond cultivar showed higher quercetin, kaempferol, and DPPH activity, especially at ZnO-NPs (5.0 mg mL^−1^) and Fe_3_O_4_-NPs (2.5–5.0 mg mL^−1^).	[105]
Drought stress (pot experiment)	ZnO-NPs	Cucumber (*Cucumis sativus* L.)	25, 100 mg L^−1^ (foliar application)	Improved growth and biomass under drought; enhanced photosynthetic pigments, photosynthesis, and PSII activity (maximal at 100 mg L^−1^); reduced ROS and lipid peroxidation; increased enzymatic and non-enzymatic antioxidants; elevated proline, glycine betaine, amino acids, and sugars; mitigated drought-induced decline in phenols and mineral nutrients.	[97]
Drought stress (60% field capacity, FC)	ZnO-NPs	Pea (*Pisum sativum* L.) common	50 ppm (seed priming), 100 ppm (foliar), 150 ppm (soil drenching)	Seed priming (50 ppm) enhanced growth, physiology, antioxidant levels, and mineral content by 35–57%. Foliar (100 ppm) enhanced by 43–64%. Soil drenching (150 ppm) improved by 47–64%. All treatments reduced osmotic stress and boosted drought resilience.	[98]
Drought stress (withholding) irrigation for 6 days at the first square stage)	ZnO-NPs vs. zinc sulfate (ZnSO_4_·7H_2_O)	Cotton (*Gossypium hirsutum* L.)	ZnO-NPs: 0.1 g Kg^−1^ potting mix; ZnSO_4_·7H_2_O: 1.0 g Kg^−1^ potting mix	ZnO-NPs improved growth, root and shoot biomass, hydration status, antioxidant enzyme activity, membrane integrity, and mineral uptake compared to ZnSO_4_. ZnO-NPs also enhanced yield and fiber quality under drought and well-watered conditions.	[99]
Drought stress (optimal irrigation = 20% depletion, moderate deficit = 50% depletion, severe deficit = 80% depletion)	ZnO-NPs alone or combined with arbuscular mycorrhizal fungi (AMF)	Soybean (*Glycine max* L.)	ZnO-NPs: 200 mg L^−1^ (foliar); AMF: proprietary blend; combined ZnO-NPs + AMF	Combined ZnO-NPs + AMF enhanced root colonization, nutrient uptake (N, P, K, Zn), proline, soluble carbohydrates, and antioxidant enzymes; reduced oxidative markers. Increased yield (+145%) and oil content (+24%), improved oil composition (higher linoleic and linolenic acids, lower palmitic and stearic acids), and oil quality indices.	[101]
Drought stress (100% irrigation requirement = control, 75% irrigation requirement = moderate stress, 50% irrigation requirement = severe stress)	ZnO-NPs compared with jasmonic acid (JA)	Sugar beet (*Beta vulgaris* L.)	ZnO-NPs: 0.2 mL L^−1^ (foliar); JA: 100 µM (foliar)	ZnO-NPs increased sugar content, beta-glycine, and antioxidant enzyme activity under drought. JA at 50% irrigation had a slightly more substantial moderating effect than ZnO-NPs. Both treatments improved root yield, antioxidant defense, and growth under water stress.	[106]
Drought stress (50% of the required moisture at the vegetative stage, maintained at 60% field capacity)	ZnO-NPs combined with melatonin (MT)	Strawberry (*Fragaria* × *ananassa* Duch)	ZnO-NPs: 0.5 g L^−1^; MT: 0.1 g L^−1^ (foliar, alone or combined)	Combined ZnO-NPs + MT improved shoot and root length, fruit biomass, bud formation, chlorophyll content, and antioxidant enzyme activity (SOD, POD, CAT), while reducing H_2_O_2_ and MDA levels under drought conditions. The combined treatment outperformed ZnO-NPs or MT alone in enhancing growth and stress tolerance.	[100]
Drought stress (irrigation withheld for 8 days until soil moisture reached 8–10% vol)	ZnO-NPs alone, fullerenol nanoparticles (FNPs), and combined ZnO + FNPs	Arabidopsis (*Arabidopsis thaliana* L.)	ZnO-NPs: 10 mg L^−1^ (foliar); FNPs: micromolar concentrations (foliar); combined ZnO + FNPs	ZnO-NPs (10 mg L^−1^) improved drought acclimation; FNPs optimized photosynthesis, stomatal conductance, and water-use efficiency due to antioxidative and hygroscopic properties; both reduced ROS, stabilized redox balance, and enhanced antioxidant enzymes. Combined ZnO + FNPs showed synergistic protective effects and modulated ABA-dependent and independent drought-response genes.	[102]
Drought stress (50% pot capacity, applied 30 days after sowing, compared with 100% PC control)	ZnO-NPs synthesized from corn husks	Wheat (*Triticum aestivum* L.)	Seed priming with ZnO-NPs (200 mg L^−1^) or DR GREEN fertilizer (40 g L^−1^)	ZnO-NP priming enhanced activity of antioxidant enzymes (POX, CAT, GR), increased total phenolics, flavonoids, and sugars; alleviated oxidative stress under drought but also showed potential phytotoxic risks; overall improved stress resilience compared to conventional nutri-priming.	[107]
Drought stress (withholding irrigation from tasseling to grain filling for 21 days)	ZnO-NPs, 100 mg L^−1^), manganese oxide nanoparticles (MnO-NPs, 20 mg L^−1^), ZnO-NPs + MnO-NPs, TNAU Nano Revive (1.0%), ZnSO_4_ 0.25% + MnSO_4_ 0.25%	Maize (*Zea mays* L.)	Foliar application of ZnO-NPs (100 mg L^−1^) and MnO-NPs (20 mg L^−1^), alone or in combination	ZnO-NPs and MnO-NPs reduced the anthesis–silking interval, improved chlorophyll index, proline, and green leaf area under drought. Combined ZnO-NPs + MnO-NPs enhanced seed-filling rate (+90%), duration (+13%), and seed yield (+52%) compared with the control.	[108]
Drought stress induced with 20% PEG solution (15 days)	Chitosan-loaded ZnO-NPs synthesized with *Nigella sativa*	Maize (*Zea mays* L.)	Foliar spray with CSNPs ranging from 300 µg L^−1^ to 500 mg L^−1^	Chitosan-loaded ZnO-NPs mitigated drought stress by enhancing growth traits (plant length +10.2%, leaf area +29.9%, ear length +8.7%, cob weight +47.2%, grains +462.4%). Improved osmotic potential, relative water content, and membrane stability index. Reduced oxidative stress markers (MDA −21.1%, proline −5.5%) while increasing proteins (+61.7%), flavonoids (+21.1%), and antioxidant enzymes (CAT +13.7%, POD +27.2%, SOD +24.7%).	[109]
Drought stress induced with 20% PEG-6000 (hydroponics) and irrigation withholding (pot experiment)	ZnO-NPs, at 60 ppm; Iron oxide nanoparticles (FeO-NPs)	Wheat (*Triticum aestivum* L., cultivars Johar-16, Faisalabad-08, Aas)	Foliar/nutrient solution application of ZnO-NPs (60 ppm) and FeO-NPs (dose not specified beyond ~58 nm characterization)	Drought reduced shoot length, RWC, and increased proline. ZnO-NPs (60 ppm) restored shoot length (up to 66.7 cm in Johar-16), RWC (88.4%), and improved root biomass. FeO-NPs enhanced shoot dry weight (9.73 g) and grain yield (+74% in Faisalabad-08). Johar-16 showed the best physiological adjustment, Faisalabad-08 showed the best yield resilience, and Aas showed moderate responses. ZnO-NPs were more effective for growth recovery, and FeO-NPs for yield enhancement.	[110]
Drought stress induced by different field capacities (80, 60, 40, and 20%) Drought stress induced by different field capacities (80, 60, 40, and 20%)	ZnO-NPs used for seed priming (nanopriming)	Wheat (*Triticum aestivum* L.)	Hydropriming; ZnO-NPs at 0.05, 0.1, 0.25, 0.5, and 1.0 g/L	Hydropriming and nanopriming alleviated drought effects, improving relative water content, leaf area, and root traits. Specific root length increased under drought but was reduced by priming, indicating that stress was alleviated. ZnO-NPs increased osmolytes (proteins, proline, soluble sugars), antioxidants (DPPH activity, phenolics), and induced a stress-resistant protein band at 40% field capacity. Nanopriming modulated protein synthesis/metabolism, enhancing drought tolerance in both cultivars.	[111]
Drought stress at 80% and 60% field capacity (FC)	ZnO-NPs (nanopriming) and bulk ZnO seed priming	Wheat (*Triticum aestivum* L.) seedlings	60 mg L^−1^ (ZnO-NPs and bulk ZnO for seed priming)	Both ZnO-NPs and bulk ZnO priming mitigated drought stress effects, especially at 60% FC. Enhanced antioxidant enzyme activities (POD ↑ 91.8–289.9% shoots, 218.6–261.6% roots), phenolics ↑ 194.4% shoots and 1139.6% roots, H_2_O_2_ scavenging ↑ 124.9–147.6%, lipid peroxidation inhibition ↑ 320.6–433%. Increased free amino acids (↑ 393.8–502.8% roots) and soluble carbohydrates (↑ 183.4% roots). Results confirmed adequate biochemical and physiological protection.	[112]
Drought stress (greenhouse; control = irrigation every 3 days; drought = irrigation withheld until wilting)	ZnO-NPs; Proline-primed ZnO (ZnOP); Betaine-primed ZnO (ZnOBt)	Tomato (*Solanum lycopersicum*)	Soil application: 50 and 100 mg kg^−1^ (mixed with 2.5 kg soil per pot)	Increased plant height (ZnOP50: 1.09 m; ZnO100: 1.06 m); improved chlorophyll content (ZnOP: +86%, ZnOBt: +87.16%); maximum yield with ZnOP (204 g/g/plant); reduced oxidative stress (lower phenolics/flavonoids in stressed leaves); enhanced fruit nutritional quality (↑ phenolics, flavonoids, lycopene, betaine, and proline).	[113]

**Table 2:** Effects of zinc oxide nanoparticles (ZnO-NPs) on plant responses under salt stress.

Abiotic Stress	Nanoparticles	Crop	Type of Application and Doses	Response Under the Effect of NPs	Reference
Salt stress (200 mM NaCl)	ZnO-NPs	Common bean (*Phaseolus vulgaris* L.)	25, 50, 100, 200 mg L^−1^ (foliar spray, seed nanopriming, soil application)	ZnO-NPs alleviated salinity stress by enhancing plant growth traits, including fresh weight (+24%), relative water content (+27%), plant height (+33%), and chlorophyll content (+37%). Stress markers such as proline (↑ >100%), Na^+^ accumulation, and ROS levels were significantly reduced. Antioxidant enzyme activities improved, supporting redox balance and membrane stability. Among the application methods, nanopriming was the most effective, restoring growth and physiological traits to near control levels.	[77]
Salt stress (50 mM and 100 mM NaCl)	ZnO-NPs	Pea (*Pisum sativum* L.)	Foliar application of ZnO-NPs at 50 and 100 ppm (applied individually and with NaCl)	ZnO-NPs at 50 ppm alone improved growth traits (root length ↑, other growth parameters ↑) and reduced oxidative damage (MDA ↓, H_2_O_2_ ↓). High salt concentrations (50–100 mM NaCl) alone decreased all parameters. The best combined treatment was 50 ppm ZnO-NPs + 50 mM NaCl, which significantly improved root length, physiological traits, and lowered MDA, glycine betaine, and H_2_O_2_.	[20]
Salt stress (150 mM NaCl, applied at 15 DAS)	ZnO-NPs	Rice (*Oryza sativa* L.)	Foliar spray of ZnO-NPs at 100 mg L^−1^ for five consecutive days (26–30 DAS)	ZnO-NPs significantly improved shoot and root growth, photosynthetic pigments (SPAD ↑ 29%), and photosynthesis (net rate ↑ 24%). Enhanced nutrient uptake (N, P, K, Zn ↑ 9–17%) and boosted antioxidant enzyme activities. ZnO-NPs reduced oxidative damage by lowering H_2_O_2_ and MDA contents, and mitigated salinity-induced proline over-accumulation. Overall, ZnO-NPs promoted growth recovery and enhanced stress tolerance under severe salt stress.	[114]
Salt stress (0, 150, and 300 mM NaCl)	ZnO-NPs and copper oxide nanoparticles (CuO-NPs)	*Radish* (*Raphanus sativus* L.)	Foliar spray at 100 mg L^−1^ for 15 days on 15-day-old seedlings	Salinity reduced nutrient uptake, leaf area, and photosynthetic efficiency, while increasing proline, anthocyanin, and flavonoid levels, as well as antioxidant enzyme activities. Foliar application of ZnO-NPs significantly alleviated these effects by improving leaf area, mineral content, photosynthetic electron transport rate, PSII quantum yield, and stomatal conductance. ZnO-NPs also reduced oxidative stress by lowering proline, anthocyanin, and flavonoid levels, as well as enzymatic activities (SOD, APX, and GOPX). ZnO-NPs were more effective than CuO-NPs in enhancing growth and mitigating salt-induced damage.	[123]
Salt stress (0, 100, 200 mM NaCl)	Green-synthesized zinc oxide nanoparticles (ZnO-NPs) and bulk Zn	Quinoa (*Chenopodium quinoa* L.)	Foliar application at 0, 50, 100, and 200 ppm	Salinity stress reduced plant growth (height, weight, diameter), chlorophyll content, and altered ion ratios (K/Na and Ca/Na), while increasing oxidative stress markers (H_2_O_2_ and MDA) and osmolytes (proline and sucrose). The application of ZnO-NPs mitigated these effects by enhancing antioxidant enzyme activities (SOD, CAT, POD), reducing oxidative damage, stabilizing ion homeostasis, and improving chlorophyll content and plant growth. ZnO-NPs were more effective than bulk Zn in promoting stress tolerance and physiological recovery.	[124]
Salt stress (field conditions, saline soil—exact NaCl level not specified)	ZnO NPs, SiO_2_ NPs, combined SiO_2_–ZnO NPs (with and without proline)	Wheat (*Triticum aestivum* L.)	Foliar spray: Control (water), Proline (Pro), SiO_2_ NPs (Si), ZnO NPs (Zn), SiO_2_–ZnO NPs (Si + Zn), Proline + SiO_2_–ZnO NPs (Pro + Si + Zn)	Exogenous application of SiO_2_–ZnO NPs, especially in combination with proline, alleviated salt stress by increasing leaf chlorophyll, proline, K, Si, Zn content, and K/Na ratio, while reducing Na accumulation. Also enhanced nutrient content in straw and grains, crude protein in grains, and significantly improved biological, grain, and straw yields under salinity stress. The best performance was achieved with Pro + SiO_2_–ZnO NPs.	[115]
Salt stress—150 mM NaCl (hydroponic)	ZnO-NPs	Cotton (*Gossypium hirsutum* L.)	Foliar spray: 50, 100, 150, 200 mg L^−1^ (10 mL per plant, front and back of leaves, seven consecutive days)	Improved shoot and root biomass, leaf area, plant height, and stem diameter; reduced MDA, H_2_O_2_, and O_2_^−^; enhanced antioxidant enzymes (SOD, POD, CAT); regulated expression of stress-related genes (↑ CNGC, NHX2, AHA3, HAK17; ↓ SKOR), stabilizing Na^+^/K^+^ ratio and improving salt tolerance.	[125]
Salt stress—150 mM NaCl	ZnO-NPs	Coffee (*Coffea arabica)*	Foliar spray: 50 and 100 mg L^−1^	Mixed effects: increased proline (33–77%) and CAT activity (69–152%); reduced H_2_O_2_ (−18.7%); but also, higher Na^+^ accumulation (+45%), increased MDA (+3–50%), and reduced carotenoids at 100 mg L^−1^, limiting photoprotection.	[126]
Salt stress—150 mM NaCl	ZnO-NPs	Maize (*Zea mays* L.)	Foliar spray, 2 g L^−1^	Improved growth and development under salinity: increased chlorophyll, enhanced fatty acid synthesis, higher protein and sugar metabolism, improved biomass (leaves, stalks, cobs, seeds), and reduced oxidative stress (lower H_2_O_2_ and MDA).	[127]
Saline–sodic stress (soil condition)	ZnO-NPs	Rice (*Oryza sativa* L.)	30 kg ha^−1^ (mixed with the topsoil)	Reduced Na^+^ and MDA levels; increased K^+^, Zn^2+^, chloroplast pigments, quantum yield, PIABS, and active PSII reaction centers; improved electron and energy transfer in photosystem; enhanced photosynthesis and resistance to saline–sodic stress.	[128]
Salinity stress (100 mM NaCl)	ZnO NPs synthesized from Phragmites karka	Tomato (*Solanum lycopersicum* L. seedlings)	Foliar/soil treatments at 20 mg L^−1^ and 50 mg L^−1^	ZnO NPs improved overall growth under salinity; 50 mg L^−1^ (T20) enhanced shoot length (3-fold) and increased nodes and internodes; 20 mg L^−1^ (T16) increased leaf number. ZnO NPs effectively promoted salt resilience by stimulating growth parameters.	[129]
Salinity stress, saline soil (EC 7.8 dS m^−1^)	ZnO-NPs	Mung bean (*Vigna radiata* L.)	Seed priming with 50, 100, 500, 1000 mg L^−1^	Improved germination %, shoot length, and shoot dry weight—increased SOD, POX, and proline, reduced lipid peroxidation and membrane injury. Low dose (50 mg L^−1^) was most effective, while 1000 mg L^−1^ negatively affected root traits in the sensitive genotype.	[118]
Salinity stress (0, 50, 100 mM NaCl)	ZnO-NPs (alone and in combination with biochar)	Spinach (*Spinacia oleracea* L.)	- Priming: 100 mg L^−1^ ZnO-NPs - Foliar spray: 100 mg L^−1^ ZnO-NPs - Biochar: 2.0% (*w*/*w*) soil amendment	Salinity (100 mM) caused the maximum reduction in growth and oxidative stress. ZnO-NPs alone improved growth, chlorophyll, gas exchange, and antioxidant activity. The combined ZnO-NPs + biochar treatment was most effective, reducing Na^+^ accumulation (−57.7% in roots, −61.3% in leaves), enhancing nutrient content, and improving nutritional quality and salinity tolerance.	[116]
Salt stress (50 mM NaCl)	Biogenic zinc oxide nanoparticles (ZnO-NPs) were synthesized using *Acacia nilotica* leaf extract	Chili (*Capsicum annuum* L.)	Foliar spray: 0, 25, 50, 75, 100 ppm	Foliar ZnO-NPs (100 ppm) significantly enhanced shoot length (+38.6%), root length (+25.5%), chlorophyll content (+23.3%), phenolics (+12.5%), and zinc accumulation (+38.7%). Also reduced oxidative stress markers: MDA (−54.4%) and H_2_O_2_ (−33.1%). Overall, 100 ppm was the most effective treatment for growth and stress tolerance.	[121]
Salt stress (100 mM NaCl)	ZnO-NPs, SiO_2_ NPs (compared to ZnSO_4_ and K_2_SiO_3_)	Maize (*Zea mays* L.)	ZnO: 10 mg L^−1^, SiO_2_: 90 mg L^−1^, applied in hydroponic Hoagland solution	ZnO-NPs increased K^+^ concentration and K^+^/Na^+^ ratio, enhancing ionic homeostasis; SiO_2_-NPs improved osmotic adjustment and limited Na^+^ accumulation. Both improved biomass, chlorophyll content, and salinity tolerance index.	[130]
Salinity stress (50 mM NaCl, 33.3 mM CaCl_2_, 25 mM NaCl + 16.6 mM CaCl_2_)	ZnO-NPs, SiO_2_ NPs	Lettuce (*Lactuca sativa*)	Foliar application, 100 mg L^−1^	Under non-saline conditions, both NPs improved growth. SiO_2_ NPs increased biomass, root architecture, and antioxidant enzymes (SOD, GR); ZnO NPs enhanced root biomass, root architecture, and leaf chlorophyll. Under CaCl_2_ stress, SiO_2_ NPs improved root growth, non-enzymatic antioxidants, and CAT, APX, and GR activity. ZnO NPs caused greater physiological damage under CaCl_2_ and NaCl + CaCl_2_ (impaired root development, reduced PSII efficiency). Overall, SiO_2_ NPs conferred partial tolerance; ZnO NPs were detrimental under combined stresses.	[31]
Salinity stress (1.0, 2.0, 3.0, 4.0, 5.0 dS m^−1^ NaCl)	ZnO-NPs	Basil (*Ocimum basilicum*)	Foliar spray, 1.5–2.0 mg L^−1^	Salinity reduced growth and photosynthesis, while increasing lipid peroxidation, electrolyte leakage (EL), and antioxidant markers. ZnO NPs (1.5–2.0 mg L^−1^) enhanced growth and photosynthetic traits, increased antioxidant activity, and reduced EL and lipid peroxidation. Most effective dose identified: 2.0 mg L^−1^.	[122]
Salinity stress 100 mM NaCl	ZnO-NPs and Salicylic Acid (SA)	Salvia varita (*Salvia virgata*)	Foliar application ZnO NPs: 20 mg L^−1^ SA: 500 μM foliar spray	Salinity decreased chlorophyll a, b, and carotenoids; increased MDA, H_2_O_2_, phenolics, flavonoids, sugars, and proline. Elicitors (SA, ZnO NPs, SA + ZnO NPs) increased pigments, proline, sugars, phenolics, and flavonoids. They boosted antioxidant enzymes (CAT, GR, APX, SOD) a synergistic effect was observed, resulting in reduced oxidative stress and improved growth under salinity conditions.	[117]
Salinity stress 50 mM and 100 mM NaCl	ZnO-NPs	Tomato (*Solanum lycopersicum* L.)	750 ppm ZnO NPs, seed priming for six h before sowing	Salinity reduced chlorophyll a, b, and carotenoids, and increased MDA, H_2_O_2_, phenolics, flavonoids, sugars, and proline. Elicitors (SA, ZnO NPs, SA + ZnO NPs) increased pigments, proline, sugars, phenolics, flavonoids, and boosted antioxidant enzymes (CAT, GR, APX, SOD). Combination NaCl + SA + ZnO NPs: ↑ proline (+21.55%), ↑ sugars (+15.73%), ↓ MDA (−42.28%), ↓ H_2_O_2_ (−42.34%). A synergistic effect was observed, resulting in reduced oxidative stress and improved growth under salinity conditions.	[119]
Salinity stress (20, 40, 80, 120 mmol L^−1^ NaCl)	ZnO-NPs ZnO bulk	Chickpea (*Cicer arietinum*)	Foliar application, 50 mg L^−1^ (ZnO bulk and ZnO NPs)	Salt stress reduced growth, chlorophyll, K^+^ and Zn^2+^, and increased Na^+^, Cl^−^, MDA, and proline. ZnO bulk and ZnO NPs enhanced growth, nutrient uptake, and antioxidant enzyme activity (SOD, CAT, APX, GPX, GR). ZnO bulk decreased MDA by 30–47% and proline by 1.6–6%; ZnO NPs decreased MDA by 31–58% and proline by 21–28%, showing stronger stress mitigation compared to bulk ZnO.	[120]

**Table 3:** Effects of zinc oxide nanoparticles (ZnO-NPs) on plant responses under heat stress.

Abiotic Stress	Nanoparticles	Crop	Type of Application and Doses	Response Under the Effect of NPs	Reference
Heat stress (heatwave, 37 °C, 6 days)	ZnO-NPs	Rice (*Oryza sativa* L.)	Foliar spray: 50, 100, 200 mg L^−1^ (0.5 mL per plant day^−1^ for 6 d); life cycle: 100 mg L^−1^ (6.7 mL per plant day^−1^)	Increased grain yield (22.1%), protein (11.8%), and amino acids (77.5%). Enhanced nutrient accumulation (Zn, Mn, Cu, Fe, Mg ↑15.8–416.9%), chlorophyll (↑22–25%), Rubisco activity (↑21.2%), and antioxidant activity (↑27–31%). Reversed transcriptomic dysregulation, improved photosynthesis (↑74.4%), and stabilized phyllosphere microbial community under HW stress.	[131]
Heat stress (field conditions, summer 2022, Peshawar valley)	ZnO-NPs	Maize (*Zea mays* L.)	Seed priming: Control, 100 mg L^−1^, 150 mg L^−1^ Foliar spray (V8 stage): Control, 100 mg L^−1^, 150 mg L^−1^	Foliar spray (150 mg L^−1^): ↑ leaf area, height, yield, Zn uptake (+53%); seed priming (150 mg L^−1^): ↑ plant height, yield, Zn uptake (+59.7%); improved chlorophyll, photosynthesis, CTD, reduced electrolyte leakage and HSPs.	[40]
Heat stress: 38 °C, 2 h day^−1^, 6 days	ZnO-NPs (biosynthesized) enriched with *Lessertia frutescens* leaf extract (CLE)	Oregano (*Origanum vulgare* L.)	Foliar spray: CLE (2%) + ZnO NPs at 25, 50, 75 mg L^−1^	CLE + ZnO NPs (50–75 mg L^−1^): ↑ growth, yield, chlorophylls, carotenoids, essential oil, phenolics, ascorbic acid, antioxidant enzymes (CAT, APX, SOD); ↓ MDA and electrolyte leakage under heat stress.	[132]
Heat stress (45 °C Day/34 °C night, 7 days)	ZnO-NPs	Alfalfa (*Medicago sativa* L.)	Foliar spray: 30, 60, 90 mg L^−1^; treatments: No heat stress (NHS), pretreatment before heat stress (BHS), post-treatment after heat stress (AHS)	ZnO-NPs (esp. 90 mg L^−1^) ↓ membrane damage, lipid peroxidation and oxidative stress; ↑ antioxidant systems and osmolytes. BHS > AHS: reversible chloroplast/mitochondria damage, improved growth and physiological performance.	[38]
Heat stress (37 °C)	ZnO-NPs	Arabidopsis *(Arabidopsis thaliana)*	1 µg mL^−1^ in growth medium	ZnO-NPs alone did not alleviate TGS-GUS, but under heat stress, they enhanced transcriptional gene silencing, showing a synergistic effect between NPs and heat stress on genomic instability, which is modulated by developmental stage and heat duration.	[133]
Heat stress (24 °C vs. 28 °C; heatwave simulation)	ZnO-NPs	(*Chlorella pyrenoidosa*)	1.0 mg L^−1^	ZnO-NPs caused growth inhibition, which was stronger at 24 °C than at 28 °C. HW (28 °C) reduced ROS and cell damage, altered algal surface properties, and decreased Zn uptake. Metabolomics revealed disturbances in amino acids, fatty acids, and energy metabolism under ZnO-NPs stress, which were mitigated under HW, thereby improving algal adaptability.	[134]
Radiation stress 60 Gy gamma irradiation	ZnO–NPs	Spinach (*Spinacia oleracea* L.)	ZnO–NPs seed priming (0, 50, 100, 200 ppm for 24 h)	Maximum germination at 100 ppm ZnO–NPs (92%) and 100 ppm + 60 Gy (90%). Highest chlorophylls and carotenoids at 100 ppm + 60 Gy. Proline peaked (1.069 mg g^−1^ FW) at 200 ppm ZnO–NPs + 60 Gy. Anatomical changes: epidermal tissue thickened, especially at 200 ppm. Molecular (SCoT) markers revealed ZnO–NPs reduced gamma-induced genetic alterations. ZnO nanoparticles act as nanoprotective agents, mitigating radiation damage.	[135]
UV-B radiation stress (30 min/day for 15 days, hydroponic culture)	ZnO-NPs	Chickpea (*Cicer arietinum* L.)	Foliar spray: 50 and 100 mg L^−1^ (before UV-B exposure)	UV-B reduced root length (−40%), shoot FW (−17%), SDW (−15%), and stem thickness (−39%). ZnO-NPs improved growth: shoot FW (+56%, +63%), SDW (+40%, +79%), shoot length (+21%, +12%). At 100 mg L^−1^, enhanced stem thickness (+31%) and vascular tissues (xylem, phloem, collenchyma). Increased TPC (+8%), TFC (+30%), and antioxidant activity (DPPH ↑15.78% at 50 mg L^−1^). Mitigated UV-B-induced anatomical and physiological damage.	[136]
Heat stress (field, El Wadi El Gadeed, Egypt)	Zn-NPs Fe-NPs	Wheat (*Triticum aestivum* L.)	Foliar spray at 0, 0.25, 0.50, 0.75, 1.0, and 10 ppm	Best performance observed at 10 ppm Zn-NPs and 0.25 ppm Fe-NPs. Enhanced yield in heat-sensitive cultivar (Gimmeza7). Increased antioxidant enzymes (GST, SOD, POX, CAT), decreased MDA (lipid peroxidation). The isozyme profile showed new bands linked to stress tolerance. Improved plant survival and yield under heat stress.	[138]
Heat stress (35/30 °C Day/night), drought stress (35% WHC), and combined drought + heat stress	Green-synthesized ZnO-NPs (Papaya fruit extract, 10 ppm) + PGPR (*Pseudomonas* sp.)	Wheat (*Triticum aestivum* L.)	Foliar application of ZnO-NPs (10 ppm), alone or in combination with PGPR	Heat and drought stresses increased MDA and H_2_O_2_, resulting in reduced growth and pigment production. ZnO-NPs + PGPR improved biomass, photosynthetic pigments, nutrients, soluble sugars, proteins, and IAA. Combination treatment enhanced proline, ABA, antioxidant enzymes (SOD, POX, CAT, APX, GR, DHAR), and reduced electrolyte leakage, MDA, and H_2_O_2_. The synergistic effect provided stronger protection under combined stress.	[139]
Heat stress: 25/20 °C (control), 35/30 °C (moderate), 40/35 °C (severe)	ZnO-NPs	Snapdragon (*Antirrhinum majus*) seedlings	0, 50, 100, 200 mg L^−1^ foliar spraying	Heat stress reduced growth, chlorophyll, and antioxidant activity. ZnO-NPs alleviated inhibition by increasing chlorophyll (↑by 3.8–7.7%), carotenoids (↑by 4.9–11.7%), soluble sugars (↑by 12.7–44.8%), and proline (↑by 18.2–32.9%). Enhanced AsA-GSH cycle enzymes: APX (↑35.3–86.3%), DHAR (↑13.5–24.6%), MDHAR (↑58.5–81.5%), GR (↑8.6–19.7%), plus higher AsA, GSH, AsA/DHA, and GSH/GSSG ratios. Reduced MDA, electrolyte leakage, O_2_•^−^, and H_2_O_2_. Best effect at 100 mg L^−1^, conferring the highest heat tolerance.	[137]

**Table 4:** Effects of zinc oxide nanoparticles (ZnO-NPs) on plant responses under heavy metal stress.

Abiotic Stress	Nanoparticles	Crop	Type of Application and Doses	Response Under the Effect of NPs	Reference
Heavy metal stress: wastewater contamination with Cadmium (Cd) and Chromium (Cr^6+^)	Biologically synthesized ZnO-NPs (from *Shewanela* sp.)	Wheat (*Triticum aestivum* L.)	Foliar spray: 0, 25, 50, 100 mg L^−1^ applied at intervals for 40 days	100 mg L^−1^ ZnO-NPs gave the best results. Cd in shoots ↓19.6%, 43.8%, 90.9% (at 25, 50, 100 mg L^−1^). Cr^6+^ in shoots ↓14.6%, 39.3%, 94.9% (at 25, 50, 100 mg L^−1^). Growth, germination, chlorophyll, total soluble sugars, total free amino acids, and ascorbic acid increased. Antioxidant enzymes (APX, SOD, POD, CAT) ↑. Oxidative stress markers (MDA, H_2_O_2_, EL, O_2_•^−^) ↓. ZnO-NPs alleviated heavy metal toxicity, improving growth and physiology.	[140]
Heavy metal stress: Cadmium (Cd) contamination	ZnO-NPs, Fe_3_O_4_-NPs, and combined NPs	Peanut (*Arachis hypogaea* L.)	Foliar spray: 50–400 mg L^−1^; optimal at 150 mg L^−1^ combined NPs	Combined NPs reduced Cd in roots (↓52.1%) and shoots (↓47.8%); biomass ↑42.9% (roots) and ↑100.2% (shoots). At 150 mg L^−1^, root Cd dropped from 0.619 to 0.245 mg g^−1^ and shoot Cd from 0.187 to 0.148 mg g^−1^. Transcriptomics: upregulated GST23, POD2 (antioxidant defense); downregulated transporters ABCC2, Nramp2, ABCG29, ABCG2 (Cd uptake ↓). Combined NPs synergistically enhanced growth, antioxidant capacity, and Cd resistance.	[141]
Heavy metal stress: Arsenic (As, 10 ppm) and Chromium (Cr, 10 ppm)	ZnO-NPs	Wheat (*Triticum aestivum* L.)	Foliar spray: 10, 20, 30 ppm ZnO-NPs, applied once after 14 days of germination	ZnO-NPs reduced As and Cr toxicity, improving morphological traits, physiology, antioxidant enzymes, and yield. Variety Faisalabad 2008 performed better than Aas 2011. ZnO-NPs decreased heavy metal accumulation, enhanced biochemical and antioxidant responses, and mitigated stress-induced yield losses. ZnO-NPs alleviated As and Cr toxicity, improving wheat growth and productivity.	[143]
Heavy metal stress: Cadmium (Cd, 19.2 mg kg^−1^ soil)	ZnO-NPs	Alfalfa (*Medicago sativa* L.)	Foliar spray: 100 mg L^−1^ ZnO-NPs	Cd stress reduced shoot height, biomass, and induced ROS accumulation, oxidative stress, and programmed cell death (PCD). ZnO-NPs enhanced antioxidant activity, cell membrane stability, osmotic homeostasis, and ultrastructural integrity, thereby reducing ROS and mitigating PCD. ZnO-NPs upregulated antioxidant enzymes and PCD-related genes, enriched pathways in cell death and porphyrin/chlorophyll metabolism. ZnO-NPs alleviated Cd toxicity by promoting redox balance and enhancing molecular defenses, thereby increasing tolerance.	[148]
Heavy metal stress: Lead (Pb, 0, 50 y 100 mg kg^−1^ soil)	ZnO-NPs, TiO_2_-NPs, ZnO + TiO_2_ NPs	Moench (*Echinacea purpurea* L.)	Foliar spray: 50 mg L^−1^ ZnO-NPs, 50 mg L^−1^ TiO_2_-NPs, and combined treatment (ZnO + TiO_2_ NPs, 50 mg L^−1^ each)	Pb stress reduced growth (height, leaf area, biomass), photosynthetic pigments, Fv/Fm, Zn and Fe content, while increasing MDA, H_2_O_2_, sugars, proline, phenols, and antioxidant enzymes (SOD, APX). NPs alleviated Pb stress: improved agronomic traits, Chl a, b, carotenoids, Zn; decreased MDA, H_2_O_2_, sugars, proline, phenols, Fe, Pb content, and antioxidant overactivation. Essential oil yield was increased under control by all NPs; combined ZnO + TiO_2_ reduced oil at 50 mg kg^−1^ Pb but enhanced it under 100 mg kg^−1^ Pb stress. GC/MS: germacrene, α-pinene, 1-pentadecene, D-myrcene identified as main oil constituents. The combined use of ZnO and TiO_2_ was the most effective strategy for mitigating Pb stress.	[150]
Heavy metal stress: Cadmium Cd^2+^, 10 μM, hydroponic system	ZnO-NPs (green-synthesized from Chlorella pyrenoidosa)	Chinese cabbage (*Brassica parachinensis* L.)	Foliar spray at 50 mg L^−1^ and 100 mg L^−1^	Cd stress reduced growth, pigments, and caused metabolic imbalance. Foliar ZnO-NPs improved plant height, root length, fresh biomass, and photosynthetic pigments. Increased Cu, Fe, Zn, and Mg levels in roots and leaves. Boosted antioxidant enzyme activities. Metabolomics: 481 metabolites detected; ZnO-NPs restored ~60% of Cd-affected metabolites (organic acids, amino acids, flavonoids, glycosides, nucleic acids, vitamins) to normal levels. Conclusion: ZnO-NPs balance ion absorption, modulate antioxidants, and restore metabolites, mitigating Cd toxicity.	[144]
Heavy metal stress: Chromium (Cr, 120 µM, hydroponic)	ZnO-NPs	Chickpea (*Cicer arietinum* L.)	Foliar application, 25 µM ZnO-NPs, applied twice at a 7-day interval	Cr stress reduced growth, gas exchange, cell viability, and increased Cr content and organic acid exudates. ZnO-NPs improved growth, enzymatic activities, proline, soluble sugars, protein, and gas exchange parameters, while reducing MDA, H_2_O_2_, Cr accumulation in roots/leaves, and organic acids. The tolerant genotype showed stronger alleviation.	[154]
Heavy metal stress: Pb, Cd, Zn, Ni (soil contamination)	ZnO-NPs and MnO_2_-NPs loaded on biochar (BC@ZnO, BC@MnO_2_)	Ryegrass (*Lolium perenne* L.)	Soil amendment with 1% pristine BC, BC@ZnO, or BC@MnO_2_	Both BC@ZnO and BC@MnO_2_ reduced HMs uptake; BC@MnO_2_ (1%) was most effective. Root DW = 1.365 g pot^−1^, Shoot DW = 4.163 g pot^−1^. Lowest shoot uptake at 1% BC@MnO_2_ (Pb: 13.176, Cd: 24.92, Zn: 32.407, Ni: 53.88 µg pot^−1^). Reduced translocation factor (TF) and bioconcentration. Ryegrass accumulated HMs primarily in its roots, demonstrating a strong phytostabilization capacity.	[152]
Heavy metal stress: Cadmium (Cd, 19.2 mg kg^−1^ soil)	ZnO-NPs + PGPR (*Klebsiella* sp.)	Indian mustard (*Brassica juncea*)	ZnO-NPs Foliar spray, 100 mg L^−1^) + PGPR 10^6^ CFU/mL (0.15 OD a 600 nm), 3–4 h seed priming	Cd stress reduced shoot height, biomass, and caused ROS accumulation → oxidative stress and programmed cell death (PCD). ZnO-NPs improved antioxidant enzyme activity, cell membrane stability, osmotic homeostasis, and ultrastructure; reduced ROS and PCD; upregulated detoxification-related genes. ZnO-NPs mitigated Cd toxicity at multiple physiological and molecular levels.	[147]
Heavy metal stress: Cadmium (Cd, 20 mg kg^−1^ soil)	ZnO-NPs + Plant Growth-Promoting Rhizobacteria (PGPR)	Wheat (*Triticum aestivum* L.)	Foliar ZnO-NPs (100 mg L^−1^) + PGPR inoculation	CD stress ↓ growth, biomass, photosynthesis, antioxidant activity; ↑ TaEIL1 expression. ZnO-NPs improved growth, pigmentation, and gas exchange in a dose-additive manner; the effect was amplified with PGPR. Combined ZnO-NPs + PGPR ↑ antioxidant enzymes (CAT +52.4%, POD +57.4%, SOD +60.1%, APX +47.4%); ↓ oxidative stress markers (MDA −47.4%, H_2_O_2_ −38.2%, EL −47.3%). Cd concentration ↓ in roots (56.3%), shoots (49.4%), and grains (59.4%). TaEIL1 upregulated under Cd stress, but downregulated by combined ZnO-NPs + PGPR, indicating a role in Cd tolerance. ZnO-NPs + PGPR alleviated Cd toxicity through modulation of antioxidant defense, Cd detoxification, and gene regulation, thereby ensuring food safety.	[155]
Heavy metal stress: Arsenic (As, 0.02 mg kg^−1^ soil) and Lead (Pb, 0.2 mg kg^−1^ soil)	ZnO-NPs	Sunflower (*Helianthus annuus* L.)	Soil application, 0.3 and 0.6 mg kg^−1^ ZnO-NPs; plants grown for 25, 35, and 45 days after emergence (DAE)	ZnO-NPs enhanced plant growth and root length (notably at 25 DAE). Roots, stems, and leaves accumulated As and Pb, with significant changes at 25 and 45 DAE. Bioconcentration factor (BCF) and translocation factor (TF) increased for Zn at 45 DAE, indicating Zn migration and hyperaccumulation. ZnO-NPs assisted phytoremediation by enhancing metal uptake and promoting sunflower’s hyperaccumulation traits. Limitation: tested under low heavy metal concentrations.	[156]
Cadmium (Cd, different concentrations prepared from Cd(NO_3_)_2_ in greenhouse soil)	ZnO-NPs (green-synthesized using Clinopodium vulgare L. extract) + Melatonin (MT)	Cabbage (*Brassica oleracea*)	Foliar aerosol: NP0 (DW), NP1 = 25 mg L^−1^ ZnO, NP2 = 50 mg L^−1^ ZnO, NP3 = 100 mg L^−1^ ZnO, and MT = 200 µM	ZnO-NPs exhibited concentration-dependent improvements in growth and chlorophyll content. 50 and 100 mg L^−1^ ZnO-NPs increased growth by 38–40%; 100 mg L^−1^ ZnO-NPs extended shoot length by 63.5% and enhanced Zn by 51% vs. control. ZnO-NPs reduced Cd content and increased Zn accumulation in roots/shoots. MT further enhanced growth, chlorophyll, and Zn uptake, while reducing soil Cd bioavailability. ZnO-NPs + MT synergistically alleviated Cd toxicity and promoted Zn biofortification.	[148]
Heavy metal stress: Cadmium (CdSO_4_, 25 μM, 14 days exposure)	Biogenic ZnO-NPs	Durum wheat (*Triticum turgidum* L.)	Foliar application of ZnO-NPs at 25 and 50 mg L^−1^	Cd stress reduced growth, caused chlorosis, oxidative stress, and Cd accumulation. ZnO-NPs increased chlorophyll and photosynthetic efficiency, enhanced S, Zn, and Fe accumulation, and reduced Cd uptake in shoots. Promoted thiol and phytochelatin production and upregulated sulfate transporter TdSultr1.3, boosting Cd detoxification. ZnO-NPs mitigated Cd toxicity and improved nutrient balance.	[153]
Heavy metal stress: Cadmium (Cd) 3.44 mg Cd/kg soil.	ZnO-NPs (moderate, ZM), TiO_2_-NPs, SiO_2_-NPs (low, SL), Fe_3_O_4_-NPs	Dandelion (*Taraxacum officinale*)	Foliar application of different NP types (doses: ZM = moderate, SL = low) 5, 25 y 100 mg L^−1^	ZnO-NPs (ZM) ↑ proline (+31.14%), ↓ MDA (−18.38%), ↑ soluble proteins (+72.95%). SiO_2_-NPs (SL) ↑ proline (+82.41%), ↑ soluble proteins (+78.50%). ZnO, TiO_2_, Fe_3_O_4_ reduced Cd accumulation (−24.58%, −17.37%, −20.21%). NPs enhanced nutrient uptake, chlorophyll content, and total phenols. Metabolomics: ↑ palmitic acid, stearic acid, D-glucopyranoside, myo-inositol. TiO_2_ and SiO_2_ significantly increase rhizosphere bacterial diversity (Proteobacteria, Chloroflexi, Firmicutes), aiding in Cd immobilization and pollutant degradation. Overall, NPs mitigated Cd stress through metabolic reprogramming and enrichment of the rhizosphere microbiome.	[151]
Heavy metal stress: Lead (Pb) 83 mg/L during germination (7 days)	ZnO-NPs	Pea (*Pisum sativum* L.)	10 mg L^−1^, applied as an amendment during germination (growth solution)	ZnO-NPs (10 mg L^−1^) effectively mitigated Pb-induced toxicity by restoring embryonic axis growth and redox balance. Treatment increased axis length (+34%) and biomass accumulation (fresh +26%, dry +23%), reduced oxidative damage (MDA −23%, H_2_O_2_ −33%, carbonyls −78%), and enhanced cell viability (+66%) while decreasing cell death (−10%). These effects were associated with a coordinated modulation of antioxidant and ROS-related enzymes, characterized by reduced CAT, POD, and GPX activities, increased APX and GR activities, and stimulation of glycolate oxidase and NADPH oxidase, indicating controlled ROS signaling rather than oxidative injury.	[146]
Heavy metal stress: Cadmium (CdCl_2_, 50 μM in hydroponic solution)	ZnO-NPs	Pepper (*Capsicum chinense*)	15 mg L^−1^, foliar spray applied twice daily (9 a.m. and 3 p.m.)	Cadmium stress severely impaired plant growth, photosynthetic performance, and root function; however, foliar application of ZnO-NPs (15 mg L^−1^) effectively alleviated Cd phytotoxicity. ZnO-NPs enhanced biomass accumulation, chlorophyll content, gas exchange, and PSII efficiency (Fv/Fm), while markedly reducing Cd accumulation in leaves (−30%) and roots (−75%). These improvements were associated with lower ROS levels (H_2_O_2_, O_2_•^−^), reduced lipid peroxidation, and the activation of key antioxidant enzymes (SOD, POD, CAT, APX, GR). In parallel, ZnO-NPs increased osmolytes, proteins, and secondary metabolites (phenols and flavonoids), contributing to improved redox homeostasis and stress tolerance.	[157]
Heavy metal stress: Lead (Pb-acetate, 250 and 500 mg L^−1^ in root zone	ZnO-NPs (green synthesized from Kiar plants)	Pepper (*Capsicum annuum* L.)	Seed priming with ZnO-NPs is most effective at 150 ppm	Lead stress impaired photosynthetic pigments, enzymatic activity, and growth in chili plants; however, seed priming with ZnO-NPs effectively mitigated Pb toxicity. At the optimal concentration (150 ppm), ZnO-NPs increased chlorophyll and carotenoids, enhanced antioxidant enzymes (peroxidase and catalase), and promoted phenolic and flavonoid accumulation. These physiological improvements translated into increased root and shoot growth, as well as higher fresh and dry biomass, demonstrating the strong potential of ZnO-NPs to alleviate Pb-induced stress through enhanced antioxidant defense and improved biomass production.	[158]
Heavy metal stress: Chromium (Cr, 100 mg kg^−1^ soil, applied as K_2_Cr_2_O_7_)	ZnO-NPs functionalized with melatonin (ZnO NPs@MT)	Tomato (*Solanum lycopersicum* L.)	Soil amendment with 50 mg kg^−1^ ZnO NPs@MT mixed in soil under Cr-contaminated conditions.	Cr stress reduced growth, photosynthesis, and increased ROS accumulation. ZnO NPs@MT significantly improved shoot length (+134.7%) and root length (+119.5%), enhanced chlorophyll content, CO_2_ assimilation, and stomatal conductance. It boosted antioxidant enzyme activities (SOD, CAT, APX, GR), increased proline and glycine betaine, and reduced MDA, H_2_O_2_, and electrolyte leakage. ZnO NPs@MT mitigated Cr toxicity, decreased cellular damage, and promoted stress tolerance.	[149]
Heavy metal stress: Cadmium (Cd, 50, 250, and 500 mg·kg–1 in soil)	ZnO-NPs (compared with bulk ZnO and ZnSO_4_)	Spinach *Spinacia oleracea* L.)	Soil amendment with ZnO-NPs, bulk ZnO, and ZnSO_4_ at 50, 250, and 500 mg·kg–1	Cd stress impaired spinach growth and increased Cd accumulation in plants and earthworms. The application of ZnO-NPs significantly improved growth traits (fresh weight, plant height, root length, and root morphology), reduced Cd concentration in roots (−77%), shoots (−75.6%), and earthworms (−82.3%). BCF-Cd decreased, with ZnO-NPs outperforming bulk ZnO and ZnSO_4_. Sequential BCR and DTPA-Cd analyses confirmed Cd immobilization. ZnO-NPs alleviated Cd toxicity, enhanced Zn bioaugmentation, and offered the most efficient and safe mitigation strategy for Cd-contaminated soils.	[159]
Heavy metal stress: Cadmium (Cd, 0.6 mM in soil solution)	ZnO-NPs (green-synthesized)	Maize (*Zea mays* L.)	Foliar application, 25 and 50 mg L^−1^ ZnO-NPs; evaluation after 21 days	Cadmium stress significantly reduced photosynthetic pigments and intensified oxidative damage in maize; however, ZnO nanoparticles effectively mitigated Cd toxicity. ZnO-NPs restored total chlorophyll and carotenoids, enhanced antioxidant enzyme activities (SOD, POD, CAT, APX), reduced ROS accumulation and lipid peroxidation, and improved primary metabolite status.	[142]

## Data Availability

There is no additional associated data with this article.

## Abbreviations

The following abbreviations are used in this manuscript:

ABA	Abscisic acid
APX	Ascorbate peroxidase
AsA	Ascorbate–glutathione cycle
AsA-GSH cycle	Ascorbate–glutathione cycle
CAT	Catalase
CKs	Cytokinins
ETR	Electron transport rate
Fv/Fm	Maximum quantum efficiency of PSII
ΦPSII (PhiPSII)	Effective quantum yield of PSII
GOGAT	Glutamate synthase
GPX	Glutathione peroxidase
GR	Glutathione reductase
GSH	Reduced glutathione
GSSG	Oxidized glutathione
HM	Heavy metals
IAA	Indole-3-acetic acid
MDA	Malondialdehyde
NR	Nitrate reductase
PGPR	Plant growth-promoting rhizobacteria
POD	Peroxidase
PSI (P700)	Photosystem I reaction center chlorophyll
PSII	Photosystem II
qP	Photochemical quenching coefficient
RCA	Rubisco activase
ROS	Reactive oxygen species
Rubisco	Ribulose-1,5-bisphosphate carboxylase/oxygenase
SOD	Superoxide dismutase
ZnO-NPs	Zinc oxide nanoparticles

## References

[1] Sánchez-Bermúdez M., Del Pozo J.C., Pernas M. (2022). Effects of combined abiotic stresses related to climate change on root growth in crops. Front. Plant Sci..

[2] Bibi F., Rahman A. (2023). An Overview of Climate Change Impacts on Agriculture and Their Mitigation Strategies. Agriculture.

[3] Yuan X., Li S., Chen J., Yu H., Yang T., Wang C., Huang S., Chen H., Ao X. (2024). Impacts of global climate change on agricultural production: A comprehensive review. Agronomy.

[4] Ahmed M., Tóth Z., Rizk R., Abdul-Hamid D., Decsi K. (2025). Investigation of Antioxidative Enzymes and Transcriptomic Analysis in Response to Foliar Application of Zinc Oxide Nanoparticles and Salinity Stress in *Solanum lycopersicum*. Agronomy.

[5] Mishra M., Afzal S., Yadav R., Singh N.K., Zarbakhsh S. (2025). Salinity stress amelioration through selenium and zinc oxide nanoparticles in rice. Sci. Rep..

[6] Rahim H.U., Ali W., Uddin M., Ahmad S., Khan K., Bibi H., Alatalo J.M. (2025). Abiotic stresses in soils, their effects on plants, and mitigation strategies: A literature review. Chem. Ecol..

[7] Azeem A., Mai W., Gul B., Rasheed A. (2025). Eco-physiological and growth responses of two halophytes to saline irrigation and soil amendments in arid conditions. BMC Plant Biol..

[8] Ullah S., Ali I. (2025). Understanding the Molecular Mechanisms of Nitrogen Assimilation in C_3_ plants under Abiotic Stress: A Mini Review. Phyton-Int. J. Exp. Bot..

[9] Wang T., Wu F., Liu H., Zhang X., Zhou Y., Zhang S., Yang P. (2025). Symbiotic Nodulation Enhances Legume Tolerance to Abiotic Stresses: Mechanisms and Perspectives. Plant Cell Environ..

[10] Zaib M., Zubair M., Aryan M., Abdullah M., Manzoor S., Masood F., Saeed S. (2023). A review on challenges and opportunities of fertilizer use efficiency and their role in sustainable agriculture with future prospects and recommendations. Curr. Res. Agric. Farm..

[11] Zhou X.-Q., Hayat Z., Zhang D.-D., Li M.-Y., Hu S., Wu Q., Cao Y.-F., Yuan Y. (2023). Zinc Oxide Nanoparticles: Synthesis, Characterization, Modification, and Applications in Food and Agriculture. Processes.

[12] Islam S. (2025). Toxicity and transport of nanoparticles in agriculture: Effects of size, coating, and aging. Front. Nanotechnol..

[13] Lv W., Geng H., Zhou B., Chen H., Yuan R., Ma C., Liu R., Xing B., Wang F. (2022). The behavior, transport, and positive regulation mechanism of ZnO nanoparticles in a plant-soil-microbe environment. Environ. Pollut..

[14] Martins N.C.T., Avellan A., Rodrigues S. (2020). Composites of biopolymers and ZnO NPs for controlled release of zinc in agricultural soils and timed delivery for maize. ACS Appl. Nano Mater..

[15] da Cruz T.N., Savassa S.M., Montanha G.S., Ishida J.K., de Almeida E., Tsai S.M., Junior J.L., Pereira de Carvalho H.W. (2019). A new glance on root-to-shoot in vivo zinc transport and time-dependent physiological effects of ZnSO4 and ZnO nanoparticles on plants. Sci. Rep..

[16] Mirakhorli T., Ardebili Z.O., Ladan-Moghadam A., Danaee E. (2021). Bulk and nanoparticles of zinc oxide exerted their beneficial effects by conferring modifications in transcription factors, histone deacetylase, carbon and nitrogen assimilation, antioxidant biomarkers, and secondary metabolism in soybean. PLoS ONE.

[17] Ahmed S., Khan M.T., Abbasi A., Haq I.U., Hina A., Mohiuddin M., Tariq M.A.U.R., Afzal M.Z., Zaman Q.U., Ng A.W.M. (2024). Characterizing stomatal attributes and photosynthetic induction in relation to biochemical changes in *Coriandrum sativum* L. by foliar-applied zinc oxide nanoparticles under drought conditions. Front. Plant Sci..

[18] Faizan M., Bhat J.A., Chen C., Alyemeni M.N., Wijaya L., Ahmad P., Yu F. (2021). Zinc oxide nanoparticles (ZnO-NPs) induce salt tolerance by improving the antioxidant system and photosynthetic machinery in tomato. Plant Physiol. Biochem..

[19] Karimian Z., Samiei L. (2023). ZnO nanoparticles efficiently enhance drought tolerance in *Dracocephalum kotschyi* through altering physiological, biochemical and elemental contents. Front. Plant Sci..

[20] Mustafa G., Chaudhari S.K., Manzoor M., Batool S., Hatami M., Hasan M. (2024). Zinc oxide nanoparticles mediated salinity stress mitigation in *Pisum sativum*: A physio-biochemical perspective. BMC Plant Biol..

[21] Mousavi S.M., Sedaghat A., Esmaeili M., Aftab T. (2024). Zinc in Plants: Biochemical Functions and Dependent Signaling. Metals and Metalloids in Plant Signaling (Signaling and Communication in Plants).

[22] Broadley M.R., White P.J., Hammond J.P., Zelko I., Lux A. (2007). Zinc in plants. New Phytol..

[23] Marschner H. (2011). Marschner’s Mineral Nutrition of Higher Plants.

[24] Thabet S.G., Alqudah A.M. (2024). Unraveling the role of nanoparticles in improving plant resilience under environmental stress condition. Plant Soil.

[25] Zulfiqar H.F., Afroze B., Shakoor S., Bhutta M.S., Ahmed M., Hassan S., Batool F., Rashid B., Hasanuzzaman M., Nahar K. (2024). Nanoparticles in Agriculture: Enhancing Crop Resilience and Productivity against Abiotic Stresses. Abiotic Stress in Crop Plants—Ecophysiological Responses and Molecular Approaches.

[26] Ijaz S., Abbasi B.A., Iqbal J., Ullah Z., Ijaz N., Shah N.H., Khan S., Ashraf Z., Murtaza G., Iqbal R. (2025). Exploring the Interaction of Nanomaterials with Crops Based on Multiple OMICS Tools. Stress-Resilient Crops: Coordinated Omics-CRISPR-Nanotechnology Strategies.

[27] Shirvani-Naghani S., Fallah S., Pokhrel L.R., Rostamnejadi A. (2024). Drought stress mitigation and improved yield in *Glycine max* through foliar application of zinc oxide nanoparticles. Sci. Rep..

[28] Hanif S., Sajjad A., Javed R., Zia M. (2024). The role of proline and betaine functionalized zinc oxide nanoparticles as drought stress regulators in *Coriandrum sativum*: An in vivo study. Discov. Plants.

[29] Raza M.A.S., Muhammad F., Farooq M., Aslam M.U., Akhter N., Toleikienė M., Abdulaziz-Binobead M., Ajmal-Ali M., Rzwan M., Iqbal R. (2025). ZnO-nanoparticles and stage-based drought tolerance in wheat (*Triticum aestivum* L.): Effect on morpho-physiology, nutrients uptake, grain yield and quality. Sci. Rep..

[30] Ahmed M., Marrez D.A., Rizk R., Zedan M., Abdul-Hamid D., Decsi K., Kovács G.P., Tóth Z. (2024). The influence of zinc oxide nanoparticles and salt stress on the morphological and some biochemical characteristics of *Solanum lycopersicum* L. plants. Plants.

[31] Lee C., Choi S., Leskovar D.I. (2025). SiO_2_ and ZnO nanoparticles and salinity stress responses in hydroponic lettuce: Selectivity, antagonism, and interactive dynamics. Front. Plant Sci..

[32] Ochoa-Chaparro E.H., Patiño-Cruz J.J., Anchondo-Páez J.C., Pérez-Álvarez S., Chávez-Mendoza C., Castruita-Esparza L.U., Márquez E.M., Sánchez E. (2025). Seed Nanopriming with ZnO and SiO_2_ Enhances Germination, Seedling Vigor, and Antioxidant Defense Under Drought Stress. Plants.

[33] Gao F., Zhang X., Zhang J., Li J., Niu T., Tang C., Wang C., Xie J. (2022). Zinc oxide nanoparticles improve lettuce (*Lactuca sativa* L.) plant tolerance to cadmium by stimulating antioxidant defense, enhancing lignin content and reducing the metal accumulation and translocation. Front. Plant Sci..

[34] Sehrish A.K., Ahmad S., Alomrani S.O., Ahmad A., Al-Ghanim K.A., Alshehri M.A., Tauqeer A., Ali S., Sarker P.K. (2024). Nutrient strengthening and lead alleviation in *Brassica napus* L. by foliar ZnO and TiO_2_-NPs modulating antioxidant system, improving photosynthetic efficiency and reducing lead uptake. Sci. Rep..

[35] Umair Hassan M., Huang G., Haider F.U., Khan T.A., Noor M.A., Luo F., Zhou Q., Yang B., Ul Haq M.I., Iqbal M.M. (2024). Application of Zinc Oxide Nanoparticles to Mitigate Cadmium Toxicity: Mechanisms and Future Prospects. Plants.

[36] Jalil S., Nazir M.M., Ali Q., Zulfiqar F., Moosa A., Altaf M.A., Zaid A., Nafees M., Yong J.W.H., Jin X. (2023). Zinc and nano zinc mediated alleviation of heavy metals and metalloids in plants: An overview. Funct. Plant Biol..

[37] Ghorbani A., Emamverdian A., Pehlivan N., Zargar M., Razavi S.M., Chen M. (2024). Nano-enabled agrochemicals: Mitigating heavy metal toxicity and enhancing crop adaptability for sustainable crop production. J. Nanobiotechnol..

[38] Kareem H.A., Saleem M.F., Saleem S., Rather S.A., Wani S.H., Siddiqui M.H., Alamri S., Kumar R., Gaikwad N.B., Guo Z. (2022). Zinc oxide nanoparticles interplay with physiological and biochemical attributes in terminal heat stress alleviation in mungbean (*Vigna radiata* L.). Front. Plant Sci..

[39] Thakur S., Asthir B., Kaur G., Kalia A., Sharma A. (2022). Zinc oxide and titanium dioxide nanoparticles influence heat stress tolerance mediated by antioxidant defense system in wheat. Cereal Res. Commun..

[40] Ahmad N., Shah S., Bayar J., Korany S.M., Ahmad U., Gul A., Khan W., Jalal A., Alsherif E.A., Ali N. (2025). Influence of zinc nanoparticles on maize productivity under heat stress caused by climate variability. Glob. NEST J..

[41] Wang Z., Wang S., Ma T., Liang Y., Huo Z., Yang F. (2023). Synthesis of Zinc Oxide Nanoparticles and Their Applications in Enhancing Plant Stress Resistance: A Review. Agronomy.

[42] Pejam F., Ardebili Z.O., Ladan-Moghadam A., Danaee E. (2021). Zinc oxide nanoparticles mediated substantial physiological and molecular changes in tomato. PLoS ONE.

[43] Ahmed M., Decsi K., Tóth Z. (2023). Different Tactics of Synthesized Zinc Oxide Nanoparticles, Homeostasis Ions, and Phytohormones as Regulators and Adaptatively Parameters to Alleviate the Adverse Effects of Salinity Stress on Plants. Life.

[44] Khalid F., Rasheed Y., Ashraf H., Asif K., Maqsood M.F., Shahbaz M., Zulfiqar I., Farhat F., Nawaz S., Ahmad M. (2025). Nanoparticle-mediated phytohormone interplay: Advancing plant resilience to abiotic stresses. J. Crop Health.

[45] Ali S., Ali B., Sajid I.A., Ahmad S., Yousaf M.A., Ulhassan Z., Zhang K., Ali S., Zhou W., Mao B. (2025). Synergistic effects of exogenous melatonin and zinc oxide nanoparticles in alleviating cobalt stress in Brassica napus: Insights from stress-related markers and antioxidant machinery. Environ. Sci. Nano.

[46] Singh K.M., Jha A.B., Dubey R.S., Sharma P. (2025). Nanoparticle-mediated mitigation of salt stress-induced oxidative damage in plants: Insights into signaling, gene expression, and antioxidant mechanisms. Environ. Sci. Nano.

[47] Munir N., Gulzar W., Abideen Z., Hasanuzzaman M., El-Keblawy A., Zhao F. (2023). Plant–Nanoparticle Interactions: Transcriptomic and Proteomic Insights. Agronomy.

[48] Majumdar S., Keller A.A. (2021). Omics to address the opportunities and challenges of nanotechnology in agriculture. Crit. Rev. Environ. Sci. Technol..

[49] Yadav P., Oraon P.K., Malik G., Singh S.P., Gupta A., Malhotra P., Mukherjee S. (2025). Emerging Research in Nanotechnology, Metagenomics, and Other Omics-Based Technologies for Rhizosphere Management and Increased Plant Growth and Productivity. Rhizosphere Engineering and Stress Resilience in Plants: Concepts and Applications.

[50] Khosrovyan A., Vodovnik M., Mortimer M. (2025). Omics approaches in environmental effect assessment of engineered nanomaterials and nanoplastics. Environ. Sci. Nano.

[51] Mortimer M., Wang Y., Holden P.A. (2021). Molecular mechanisms of nanomaterial-bacterial interactions revealed by omics—The role of nanomaterial effect level. Front. Bioeng. Biotechnol..

[52] Muhammad I., Shalmani A., Ali M., Yang Q.H., Ahmad H., Li F.B. (2021). Mechanisms regulating the dynamics of photosynthesis under abiotic stresses. Front. Plant Sci..

[53] Wang Z., Li G., Sun H., Ma L., Guo Y., Zhao Z., Gao H., Mei L. (2018). Effects of drought stress on photosynthesis and photosynthetic electron transport chain in young apple tree leaves. Biol. Open.

[54] Sachdev S., Ansari S.A., Ansari M.I., Fujita M., Hasanuzzaman M. (2021). Abiotic Stress and Reactive Oxygen Species: Generation, Signaling, and Defense Mechanisms. Antioxidants.

[55] Singh A., Rajput V.D., Lalotra S., Agrawal S., Ghazaryan K., Singh J., Minkina T., Rajput P., Mandzhieva S., Alexiou A. (2024). Zinc oxide nanoparticles influence on plant tolerance to salinity stress: Insights into physiological, biochemical, and molecular responses. Environ. Geochem. Health.

[56] Abdulraheem M.I., Xiong Y., Moshood A.Y., Cadenas-Pliego G., Zhang H., Hu J. (2024). Mechanisms of Plant Epigenetic Regulation in Response to Plant Stress: Recent Discoveries and Implications. Plants.

[57] Kumar M., Rani K. (2023). Epigenomics in stress tolerance of plants under the climate change. Mol. Biol. Rep..

[58] Zhou W., Wang M., Wang L., Liu Y., Tian Z., Xie L., Wang Y. (2025). Epigenetics in Plant Response to Climate Change. Biology.

[59] Kareem H.A., Hassan M.U., Zain M., Irshad A., Shakoor N., Saleem S., Niu J., Skalicky M., Chen Z., Guo Z. (2022). Nanosized zinc oxide (n-ZnO) particles pretreatment to alfalfa seedlings alleviate heat-induced morpho-physiological and ultrastructural damages. Environ. Pollut..

[60] Faseela P., Sinisha A.K., Brestič M., Puthur J.T. (2020). Chlorophyll a fluorescence parameters as indicators of a particular abiotic stress in rice. Photosynthetica.

[61] Yin M., Wang S., Wang Y., Wei R., Liang Y., Zuo L., Huo M., Huang Z., Lang J., Zhao X. (2024). Impact of Abiotic Stress on Rice and the Role of DNA Methylation in Stress Response Mechanisms. Plants.

[62] Jin Q., Chachar M., Ali A., Chachar Z., Zhang P., Riaz A., Ahmed N., Chachar S. (2024). Epigenetic Regulation for Heat Stress Adaptation in Plants: New Horizons for Crop Improvement under Climate Change. Agronomy.

[63] Mishra N., Jiang C., Chen L., Paul A., Chatterjee A., Shen G. (2023). Achieving abiotic stress tolerance in plants through antioxidative defense mechanisms. Front. Plant Sci..

[64] Rajput V.D., Harish, Singh R.K., Verma K.K., Sharma L., Quiroz-Figueroa F.R., Meena M., Gour V.S., Minkina T., Sushkova S. (2021). Recent Developments in Enzymatic Antioxidant Defence Mechanism in Plants with Special Reference to Abiotic Stress. Biology.

[65] Li P., Xia Y., Song K., Liu D. (2024). The Impact of Nanomaterials on Photosynthesis and Antioxidant Mechanisms in Gramineae Plants: Research Progress and Future Prospects. Plants.

[66] Dimkpa C.O., Andrews J., Fugice J., Singh U., Bindraban P.S., Elmer W.H., Gardea-Torresdey J., White J.C. (2020). Facile coating of urea with low-dose ZnO nanoparticles promotes wheat performance and enhances Zn uptake under drought stress. Front. Plant Sci..

[67] Dimkpa C.O., Bindraban P.S. (2017). Nanofertilizers: New products for the industry?. J. Agric. Food Chem..

[68] Rico C.M., Majumdar S., Duarte-Gardea M., Peralta-Videa J.R., Gardea-Torresdey J.L. (2011). Interaction of nanoparticles with edible plants and their possible implications in the food chain. J. Agric. Food Chem..

[69] Tripathi D.K., Singh S., Singh S., Pandey R., Singh V.P., Sharma N.C., Prasad S.M., Dubey N.K., Chauhan D.K. (2017). An overview on manufactured nanoparticles in plants: Uptake, translocation, accumulation and phytotoxicity. Plant Physiol. Biochem..

[70] Ma X., Geiser-Lee J., Deng Y., Kolmakov A. (2010). Interactions between engineered nanoparticles (ENPs) and plants: Phytotoxicity, uptake and accumulation. Sci. Total Environ..

[71] Mi K., Yuan X., Wang Q., Dun C., Wang R., Yang S., Yang Y., Zhang H., Zhang H. (2023). Zinc oxide nanoparticles enhanced rice yield, quality, and zinc content of edible grain fraction synergistically. Front. Plant Sci..

[72] Wang Y., Hui W., Zhang D., Chen X., Wang R., Xu Y., Wang L., He G. (2025). Absorption and transport of different ZnO nanoparticles sizes in Agrostis stolonifera: Impacts on physiological, biochemical responses, root exudation, and microbial community structure. Plant Physiol. Biochem..

[73] Kumar V., Naik I.S., Das B., Singh A., Nayak P., Mohapatra C., Debnath D., Tripathy M., Behera K., Masika F.B. (2025). Nanoparticles in plant system: A comprehensive review on their role in diverse stress management and phytohormone signaling. Plant Stress.

[74] Abbas Q., Yousaf B., Ali M.U., Munir M.A.M., El-Naggar A., Rinklebe J., Naushad M. (2020). Transformation pathways and fate of engineered nanoparticles (ENPs) in distinct interactive environmental compartments: A review. Environ. Int..

[75] Berwal M.K., Kumar R., Prakash K., Rai G.K., Hebbar K.B. (2021). Antioxidant defense system in plants against abiotic stress. Abiotic Stress Tolerance Mechanisms in Plants.

[76] Rao M.J., Duan M., Zhou C., Jiao J., Cheng P., Yang L., Wei W., Shen Q., Ji P., Yang Y. (2025). Antioxidant Defense System in Plants: Reactive Oxygen Species Production, Signaling, and Scavenging During Abiotic Stress-Induced Oxidative Damage. Horticulturae.

[77] Gupta A., Bharati R., Kubes J., Popelkova D., Praus L., Yang X., Severova L., Skalicky M., Brestic M. (2024). Zinc oxide nanoparticles application alleviates salinity stress by modulating plant growth, biochemical attributes and nutrient homeostasis in *Phaseolus vulgaris* L.. Front. Plant Sci..

[78] Elshoky H.A., Yotsova E., Farghali M.A., Farroh K.Y., El-Sayed K., Elzorkany H.E., Rashkov G., Dobrikova A., Borisova P., Stefanov M. (2021). Impact of foliar aerosol of zinc oxide nanoparticles on the photosynthesis of *Pisum sativum* L. under salt stress. Plant Physiol. Biochem..

[79] Seleiman M.F., Ahmad A., Alhammad B.A., Tola E. (2023). Exogenous Application of Zinc Oxide Nanoparticles Improved Antioxidants, Photosynthetic, and Yield Traits in Salt-Stressed Maize. Agronomy.

[80] Rasouli F., Asadi M., Hassanpouraghdam M.B., Aazami M.A., Ebrahimzadeh A., Kakaei K., Dokoupil L., Mlcek J. (2022). Foliar Application of ZnO-NPs Influences Chlorophyll Fluorescence and Antioxidants Pool in *Capsicum annum* L. under Salinity. Horticulturae.

[81] Azarin K., Usatov A., Minkina T., Duplii N., Kasyanova A., Fedorenko A., Khachumov V., Mandzhieva S., Rajput V.D. (2023). Effects of bulk and nano-ZnO particles on functioning of photosynthetic apparatus in barley (*Hordeum vulgare* L.). Environ. Res..

[82] Farhan M., Sathish M., Kiran R., Mushtaq A., Baazeem A., Hasnain A., Hakim F., Hassan Naqvi S.A., Mubeen M., Iftikhar Y. (2024). Plant Nitrogen Metabolism: Balancing Resilience to Nutritional Stress and Abiotic Challenges. Phyton-Int. J. Exp. Bot..

[83] Silva S., Dias M.C., Silva A.M.S. (2022). Titanium and Zinc Based Nanomaterials in Agriculture: A Promising Approach to Deal with (A)biotic Stresses?. Toxics.

[84] Dey A., Sadhukhan A. (2024). Molecular mechanisms of plant productivity enhancement by nano fertilizers for sustainable agriculture. Plant Mol. Biol..

[85] Singhal R.K., Fahad S., Kumar P., Choyal P., Javed T., Jinger D., Singh P., Saha D., MD P., Bose B. (2023). Beneficial elements: New Players in improving nutrient use efficiency and abiotic stress tolerance. Plant Growth Regul..

[86] Tripathi D., Singh M., Pandey-Rai S. (2022). Crosstalk of nanoparticles and phytohormones regulate plant growth and metabolism under abiotic and biotic stress. Plant Stress.

[87] Fatima A., Safdar N., Ain N.U., Yasmin A., Chaudhry G.E.S. (2023). Abscisic acid-loaded ZnO nanoparticles as drought tolerance inducers in *Zea mays* L. with physiological and biochemical attributes. J. Plant Growth Regul..

[88] Karabulut F. (2024). The impact of nanoparticles on plant growth, development, and stress tolerance through regulating phytohormones. Nanoparticles in Plant Biotic Stress Management.

[89] Vankova R., Landa P., Podlipna R., Dobrev P.I., Prerostova S., Langhansova L., Gaudinova A., Motkova K., Knirsch V., Vanek T. (2017). ZnO nanoparticle effects on hormonal pools in *Arabidopsis thaliana*. Sci. Total Environ..

[90] Amer H.E. (2024). Green Synthesized ZnO Nanoparticles Improve the Growth and Phytohormones Biosynthesis and Modulate the Expression of Resistance Genes in *Phaseolus vulgaris*. Egypt. J. Bot..

[91] Sonkar S., Sharma L., Singh R.K., Pandey B., Rathore S.S., Singh A.K., Porwal P., Singh S.P. (2021). Plant stress hormones nanobiotechnology. Nanobiotechnology: Mitigation of Abiotic Stress in Plants.

[92] Azhar B.J., Noor A., Zulfiqar A., Zeenat A., Ahmad S., Chishti I., Abbas Z., Shakeel S.N. (2021). Effect of ZnO, SiO_2_ and composite nanoparticles on *Arabidopsis thaliana* and involvement of ethylene and cytokinin signaling pathways. Pak. J. Bot..

[93] Sun L., Wang Y., Wang R., Wang R., Zhang P., Ju Q., Xu J. (2020). Physiological, transcriptomic, and metabolomic analyses reveal zinc oxide nanoparticles modulate plant growth in tomato. Environ. Sci. Nano.

[94] Sohail, Sawati L., Ferrari E., Stierhof Y.D., Kemmerling B., Mashwani Z.U.R. (2022). Molecular effects of biogenic zinc nanoparticles on the growth and development of *Brassica napus* L. revealed by proteomics and transcriptomics. Front. Plant Sci..

[95] Semida W.M., Abdelkhalik A., Mohamed G.F., Abd El-Mageed T.A., Abd El-Mageed S.A., Rady M.M., Ali E.F. (2021). Foliar application of zinc oxide nanoparticles promotes drought stress tolerance in eggplant (*Solanum melongena* L.). Plants.

[96] El-Zohri M., Al-Wadaani N.A., Bafeel S.O. (2021). Foliar sprayed green zinc oxide nanoparticles mitigate drought-induced oxidative stress in tomato. Plants.

[97] Ghani M.I., Saleem S., Rather S.A., Rehmani M.S., Alamri S., Rajput V.D., Kalaji H.M., Saleem N., Sial T.A., Liu M. (2022). Foliar application of zinc oxide nanoparticles: An effective strategy to mitigate drought stress in cucumber seedling by modulating antioxidant defense system and osmolytes accumulation. Chemosphere.

[98] Ishfaq A., Haidri I., Shafqat U., Khan I., Iqbal M., Mahmood F., Hassan M.U. (2025). Impact of biogenic zinc oxide nanoparticles on physiological and biochemical attributes of pea (*Pisum sativum* L.) under drought stress. Physiol. Mol. Biol. Plants.

[99] Anik T.R., Mostofa M.G., Rahman M.M., Keya S.S., Van Ha C., Khan M.A.R., Abdelrahman M., Dao M.N.K., Chu H.D., Tran L.S.P. (2025). Comparative effects of ZnSO_4_ and ZnO-NPs in improving cotton growth and yield under drought stress at early reproductive stage. Plant Sci..

[100] Anwar T., Safdar A., Qureshi H., Siddiqi E.H., Ullah N., Naseem M.T., Soufan W. (2025). Synergistic effects of *Vachellia nilotica*-derived zinc oxide nanoparticles and melatonin on drought tolerance in *Fragaria* × *ananassa*. BMC Plant Biol..

[101] Haghaninia M., Mashhouri S.M., Najafifar A., Soleimani F., Wu Q.S. (2025). Combined effects of zinc oxide nanoparticles and arbuscular mycorrhizal fungi on soybean yield, oil quality, and biochemical responses under drought stress. Future Foods.

[102] Joksimović A., Arsenov D., Borišev M., Djordjević A., Župunski M., Borišev I. (2025). Foliar application of fullerenol and zinc oxide nanoparticles improves stress resilience in drought-sensitive *Arabidopsis thaliana*. PLoS ONE.

[103] Foroutan L., Solouki M., Abdossi V., Fakheri B.A., Mahdinezhad N., Gholamipourfard K., Safarzaei A. (2019). The effects of Zinc oxide nanoparticles on drought stress in *Moringa peregrina* populations. Int. J. Basic Sci. Med..

[104] Guzel Deger A., Çevik S., Kahraman O., Turunc E., Yakin A., Binzet R. (2025). Effects of green and chemically synthesized ZnO nanoparticles on *Capsicum annuum* under drought stress. Acta Physiol. Plant..

[105] Sallam A.R., Mahdi A.A., Farroh K.Y. (2022). Improving drought stress tolerance in potato (*Solanum tuberosum* L.) using magnetite and zinc oxide nanoparticles. Plant Cell Biotechnol. Mol. Biol..

[106] Hamze H., Khalili M., Mir-Shafiee Z., Nasiri J. (2025). Integrated biomarker response version 2 (IBRv2)-Assisted examination to scrutinize foliar application of jasmonic acid (JA) and zinc oxide nanoparticles (ZnO NPs) toward mitigating drought stress in sugar beet. J. Plant Growth Regul..

[107] Rizk R., Ahmed M., Abdul-Hamid D., Zedan M., Tóth Z., Decsi K. (2025). Resulting Key Physiological Changes in *Triticum aestivum* L. Plants Under Drought Conditions After Priming the Seeds with Conventional Fertilizer and Greenly Synthesized Zinc Oxide Nanoparticles from Corn Wastes. Agronomy.

[108] Kathirvelan P., Vaishnavi S., Manivannan V., Djanaguiraman M., Thiyageshwari S., Parasuraman P., Kalarani M.K. (2025). Response of Maize (*Zea mays* L.) to Foliar-Applied Nanoparticles of Zinc Oxide and Manganese Oxide Under Drought Stress. Plants.

[109] Inam A., Javad S., Naseer I., Alam P., Almutairi Z.M., Faizan M., Zauq S., Shah A.A. (2024). Efficacy of chitosan loaded zinc oxide nanoparticles in alleviating the drastic effects of drought from corn crop. Plant Stress.

[110] Fatima S., Fatima M., Fatima T., Sarfraz A., Qureshi H., Anwar T., El-Beltagi H.S., Ismoilov I., Tukhtaboeva F., Rebouh N.Y. (2025). Enhancing drought tolerance in wheat using zinc and iron nanoparticles: Implications for sustainable industrial crop productivity. Ind. Crops Prod..

[111] Megahed S.M., El-Bakatoushi R.F., Amin A.W., El-Sadek L.M., Migahid M.M. (2025). Nanopriming as an approach to induce tolerance against drought stress in wheat cultivars. Cereal Res. Commun..

[112] El-Shazoly R.M., Othman A.A., Zaheer M.S., Al-Hossainy A.F., Abdel-Wahab D.A. (2025). Zinc oxide seed priming enhances drought tolerance in wheat seedlings by improving antioxidant activity and osmoprotection. Sci. Rep..

[113] Hanif S., Farooq S., Kiani M.Z., Zia M. (2024). Surface modified ZnO NPs by betaine and proline build up tomato plants against drought stress and increase fruit nutritional quality. Chemosphere.

[114] Dogan Y., Alam P., Sultan H., Sharma R., Soysal S., Baran M.F., Faizan M. (2025). Zinc oxide nanoparticles for sustainable agriculture: A tool to combat salinity stress in rice (*Oryza sativa*) by modulating the nutritional profile and redox homeostasis mechanisms. J. Agric. Food Res..

[115] Abd-Elzaher M.A., El-Desoky M.A., Khalil F.A., Eissa M.A., Amin A.E.E.A. (2025). Exogenously applied proline with silicon and zinc nanoparticles to mitigate salt stress in wheat plants grown on saline soil. J. Plant Nutr..

[116] Ahmad S., Khan Sehrish A., Hussain A., Zhang L., Owdah Alomrani S., Ahmad A., Al Ghanim K.A., Alshehri M.S., Ali S., Sarker P.K. (2024). Salt stress amelioration and nutrient strengthening in spinach (*Spinacia oleracea* L.) via biochar amendment and zinc fortification: Seed priming versus foliar application. Sci. Rep..

[117] Bozaba T.O., Kuru İ.S. (2024). The effect of the combined application of elicitors to *Salvia virgata* Jacq. under salinity stress on physiological and antioxidant defense. BMC Plant Biol..

[118] Sherpa D., Kumar S., Mishra S. (2024). Response of different doses of zinc oxide nanoparticles in early growth of mung bean seedlings to seed priming under salinity stress condition. Legume Res.-Int. J..

[119] Ghosh T., Yadav S.K., Choudhary R., Rao D., Sushma M.K., Mandal A., Hussain Z., Minkina T., Rajput V.D., Yadav S. (2024). Effect of zinc oxide nanoparticle based seed priming for enhancing seed vigour and physio-biochemical quality of tomato seedlings under salinity stress. Russ. J. Plant Physiol..

[120] Alabdallah N.M. (2025). Salt stress mitigation in chickpea seedlings: A comparative study of zinc oxide nano and bulk particles. Plant Soil Environ..

[121] Adnan M., Mahmood F., Zhao Z., Khaliq H., Usman M., Muhammad T., Ashraf G.A. (2025). Effect of the foliar application of biogenic-ZnO nanoparticles on physio-chemical analysis of chilli (*Capsicum annum* L.) in a salt stress environment. Environ. Sci. Adv..

[122] Hasan S.A., Khan A., Irfan M. (2024). Role of Zinc Oxide Nanoparticles in Alleviating Sodium Chloride-Induced Salt Stress in Sweet Basil (*Ocimum basilicum* L.). J. Appl. Biol. Biotechnol..

[123] Mahawar L., Živčák M., Barboricova M., Kovár M., Filaček A., Ferencova J., Visoká D.M., Brestič M. (2024). Effect of copper oxide and zinc oxide nanoparticles on photosynthesis and physiology of *Raphanus sativus* L. under salinity stress. Plant Physiol. Biochem..

[124] Türkoğlu A., Haliloğlu K., Ekinci M., Turan M., Yildirim E., Öztürk H.İ., Stansluos A.A.L., Nadaroğlu H., Piekutowska M., Niedbała G. (2024). Zinc Oxide Nanoparticles: An Influential Element in Alleviating Salt Stress in Quinoa (*Chenopodium quinoa* L. Cv Atlas). Agronomy.

[125] Qian J., Shan R., Shi Y., Li H., Xue L., Song Y., Zhao T., Zhu S., Chen J., Jiang M. (2024). Zinc Oxide Nanoparticles Alleviate Salt Stress in Cotton (*Gossypium hirsutum* L.) by Adjusting Na^+^/K^+^ Ratio and Antioxidative Ability. Life.

[126] Meléndez-Mori J.B., Lapiz-Culqui Y.K., Huaman-Huaman E., Zuta-Puscan M., Oliva-Cruz M. (2025). Can Zinc Oxide Nanoparticles Alleviate the Adverse Effects of Salinity Stress in *Coffea arabica*?. Agronomy.

[127] Ahmed M., Marrez D.A., Rizk R., Abdul-Hamid D., Tóth Z., Decsi K. (2024). Interventional Effect of Zinc Oxide Nanoparticles with *Zea mays* L. Plants When Compensating Irrigation Using Saline Water. Nanomaterials.

[128] Dang K., Wang Y., Tian H., Bai J., Cheng X., Guo L., Zhang Q., Geng Y., Shao X. (2024). Impact of ZnO NPs on photosynthesis in rice leaves plants grown in saline-sodic soil. Sci. Rep..

[129] Hanif M., Munir N., Abideen Z., Yong J.W.H., El-Keblawy A., El-Sheikh M.A. (2024). Synthesis and optimization of nanoparticles from *Phragmites karka* improves tomato growth and salinity resilience. Biocatal. Agric. Biotechnol..

[130] Shoukat A., Pitann B., Hossain M.S., Saqib Z.A., Nawaz A., Mühling K.H. (2024). Zinc and silicon fertilizers in conventional and nano-forms: Mitigating salinity effects in maize (*Zea mays* L.). J. Plant Nutr. Soil Sci..

[131] Guo S., Hu X., Wang Z., Yu F., Hou X., Xing B. (2024). Zinc oxide nanoparticles cooperate with the phyllosphere to promote grain yield and nutritional quality of rice under heatwave stress. Proc. Natl. Acad. Sci. USA.

[132] Buthelezi D. *Lessertia frutescens* L. Leaf Extract Enriched with Biosynthesized Zinc Oxide Nanoparticles Enhances the Growth, Essential Oil and Antioxidant Activities of *Origanum vulgare* L. Under Heat Stress. Proceedings of the Plants 2025: From Seeds to Food Security.

[133] Wu J., Wang T. (2020). Synergistic effect of zinc oxide nanoparticles and heat stress on the alleviation of transcriptional gene silencing in *Arabidopsis thaliana*. Bull. Environ. Contam. Toxicol..

[134] Huang K., Zeng H., Zhou Q. (2025). Heatwave enhance the adaptability of *Chlorella pyrenoidosa* to zinc oxide nanoparticles: Regulation of interfacial interactions and metabolic mechanisms. Water Res..

[135] Aly A.A., Safwat G., Eliwa N.E., Eltawil A.H., Abd El-Aziz M.H. (2023). Changes in morphological traits, anatomical and molecular alterations caused by gamma-rays and zinc oxide nanoparticles in spinach (*Spinacia oleracea* L.) plant. BioMetals.

[136] Safshekan S., Pourakbar L., Rahmani F. (2025). The effect of Zn NPs on some growth, biochemical and anatomical factors of *Chickpea* plant stem under UVB irradiation. Plant Nano Biol..

[137] Liu D., Mao F., Bai J. (2024). Effect of Different Concentrations of Exogenous ZnONPs on the Heat Tolerance of *Snapdragon*. J. Henan Agric. Sci..

[138] Hassan N.S., Salah El Din T.A., Hendawey M.H., Borai I.H., Mahdi A.A. (2018). Magnetite and zinc oxide nanoparticles alleviated heat stress in wheat plants. Curr. Nanomater..

[139] Azmat A., Tanveer Y., Yasmin H., Hassan M.N., Shahzad A., Reddy M., Ahmad A. (2022). Coactive role of zinc oxide nanoparticles and plant growth promoting rhizobacteria for mitigation of synchronized effects of heat and drought stress in wheat plants. Chemosphere.

[140] Haidri I., Ishfaq A., Shahid M., Hussain S., Shahzad T., Shafqat U., Mustafa S., Mahmood F. (2024). Enhancement of antioxidants’ enzymatic activity in the wheat crop by *Shewanela* sp. mediated zinc oxide nanoparticles against heavy metals contaminated wastewater. J. Soil Sci. Plant Nutr..

[141] Wang H., Hao C., Chen L., Liu D. (2025). Comparative physiological and transcriptomic analyses reveal enhanced mitigation of cadmium stress in peanut by combined Fe_3_O_4_/ZnO nanoparticles. J. Hazard. Mater..

[142] Hussain M., Kaousar R., Haq S.I.U., Shan C., Wang G., Rafique N., Shizhou W., Lan Y. (2024). Zinc-oxide nanoparticles ameliorated the phytotoxic hazards of cadmium toxicity in maize plants by regulating primary metabolites and antioxidants activity. Front. Plant Sci..

[143] Iqbal I., Bhatti K.H., Rashid A., Hussain K., Nawaz K., Fatima N., Hanif A. (2024). Alleviation of heavy metal toxicity (arsenic and chromium) from morphological, biochemical and antioxidant enzyme assay of wheat (*Triticum aestivum* L.) using zinc-oxide nanoparticles (ZnO-NPS). Pak. J. Bot.

[144] Ali S., Bai Y., Zhang J., Zada S., Khan N., Hu Z., Tang Y. (2024). Discovering Nature’s shield: Metabolomic insights into green zinc oxide nanoparticles Safeguarding *Brassica parachinensis* L. from cadmium stress. Plant Physiol. Biochem..

[145] Chen Z., Feng Y., Guo Z., Han M., Yan X. (2024). Zinc oxide nanoparticles alleviate cadmium toxicity and promote tolerance by modulating programmed cell death in alfalfa (*Medicago sativa* L.). J. Hazard. Mater..

[146] Kadri O., Dimkpa C.O., Chaoui A., Kouki A., Amara A.B.H., Karmous I. (2024). Zinc oxide nanoparticles at low dose mitigate lead toxicity in pea (*Pisum sativum* L.) seeds during germination by modulating metabolic and cellular defense systems. J. Agric. Food Res..

[147] Shah A.A., Zafar S., Usman S., Javad S., Aslam M., Noreen Z., Elansary H.O., Almutari K.F., Ahmad A. (2024). Zinc oxide nanoparticles and *Klebsiella* sp. SBP-8 alleviates chromium toxicity in *Brassica juncea* by regulation of antioxidant capacity, osmolyte production, nutritional content and reduction in chromium adsorption. Plant Physiol. Biochem..

[148] Anwar T., Qureshi H., Yasmeen F., Hanif A., Siddiqi E.H., Anwaar S., Gul S., Ashraf T., Okla M.K., Adil M.F. (2024). Amelioration of cadmium stress by supplementation of melatonin and ZnO-nanoparticles through physiochemical adjustments in *Brassica oleracea* var. capitata. Sci. Hortic..

[149] Sharma S., Raja V., Bhat A.H., Kumar N., Alsahli A.A., Ahmad P. (2025). Innovative strategies for alleviating chromium toxicity in tomato plants using melatonin functionalized zinc oxide nanoparticles. Sci. Hortic..

[150] Panahirad S., Dadpour M., Gohari G., Fotopoulos V. (2024). Simultaneous application of titanium dioxide (TiO_2_) and zinc oxide (ZnO) nanoparticles ameliorates lead (Pb) stress effects in medicinal plant *Echinacea purpurea* (L.) Moench. Plant Stress.

[151] Du X., Li X., Yang M., Wang T., Du Q., Gao Y., Liu J., Guo X., Tang Z. (2025). Foliar nanoparticles alleviated cadmium-induced phytotoxicity in dandelion via regulation of ionomics, metabolomics, and rhizobacterial networks. J. Hazard. Mater..

[152] Ghandali M.V., Safarzadeh S., Ghasemi-Fasaei R., Zeinali S. (2024). Heavy metals immobilization and bioavailability in multi-metal contaminated soil under ryegrass cultivation as affected by ZnO and MnO_2_ nanoparticle-modified biochar. Sci. Rep..

[153] Coppa E., Quagliata G., Palombieri S., Iavarone C., Sestili F., Del Buono D., Astolfi S. (2024). Biogenic ZnO Nanoparticles Effectively Alleviate Cadmium-Induced Stress in Durum Wheat (*Triticum durum* Desf.) Plants. Environments.

[154] Singh D., Sharma N.L., Singh D., Siddiqui M.H., Sarkar S.K., Rathore A., Prasad S.K., Gaafar A.-R.Z., Hussain S. (2024). Zinc oxide nanoparticles alleviate chromium-induced oxidative stress by modulating physio-biochemical aspects and organic acids in chickpea (*Cicer arietinum* L.). Plant Physiol. Biochem..

[155] Sehrish A.K., Ahmad S., Nafees M., Mahmood Z., Ali S., Du W., Naeem M.K., Guo H. (2024). Alleviated cadmium toxicity in wheat (*Triticum aestivum* L.) by the coactive role of zinc oxide nanoparticles and plant growth promoting rhizobacteria on TaEIL1 gene expression, biochemical and physiological changes. Chemosphere.

[156] Pérez-Hernández H., Pérez-Moreno A.Y., Méndez-López A., Fernández-Luqueño F. (2024). Effect of ZnO nanoparticles during the process of phytoremediation of soil contaminated with As and Pb cultivated with sunflower (*Helianthus annuus* L.). Int. J. Environ. Res..

[157] Tahira S., Bahadur S., Lu X., Liu J., Wang Z. (2025). ZnONPs alleviate cadmium toxicity in pepper by reducing oxidative damage. J. Environ. Manag..

[158] Mushtaq R., Shafiq M., Batool A., Din M.I., Sami A. (2024). Investigate the impact of zinc oxide nanoparticles under lead toxicity on Chilli (*Capsicum annuum* L). Bull. Biol. Allied Sci. Res..

[159] Zhang H., Zhou Q., Liu R., Zhao Z., Liu J., Siddique K.H., Mao H. (2024). Enhancing zinc biofortification and mitigating cadmium toxicity in soil–earthworm–spinach systems using different zinc sources. J. Hazard. Mater..

[160] Rastogi A., Zivcak M., Sytar O., Kalaji H.M., He X., Mbarki S., Brestic M. (2017). Impact of metal and metal oxide nanoparticles on plants: A critical review. Front. Chem..

[161] Ghaffari Yaichi Z., Hassanpouraghdam M.B., Rasouli F., Aazami M.A., Vojodi Mehrabani L., Jabbari S.F., Asadi M., Esfandiari E., Jimenez-Becker S. (2025). Zinc oxide nanoparticles foliar use and arbuscular mycorrhiza inoculation retrieved salinity tolerance in *Dracocephalum moldavica* L. by modulating growth responses and essential oil constituents. Sci. Rep..

[162] Raja V., Singh K., Qadir S.U., Singh J., Kim K.H. (2024). Alleviation of cadmium-induced oxidative damage through application of zinc oxide nanoparticles and strigolactones in *Solanum lycopersicum* L.. Environ. Sci. Nano.

[163] Hassan M.U., Kareem H.A., Hussain S., Guo Z., Niu J., Roy M., Saleem S., Wang Q. (2023). Enhanced salinity tolerance in Alfalfa through foliar nano-zinc oxide application: Mechanistic insights and potential agricultural applications. Rhizosphere.

[164] Foyer C.H., Noctor G. (2011). Ascorbate and glutathione: The heart of the redox hub. Plant Physiol..

